# Circulating biomarkers of bronchoalveolar injury help predict the need for mechanical ventilation in patients with moderate to severe COVID-19 pneumonia: A prospective cohort study

**DOI:** 10.1371/journal.pone.0337792

**Published:** 2026-06-29

**Authors:** Jérôme Allardet-Servent, Nathalie Hezard, Christel Pissier, Nathalie Bardin, Frédéric Cohen, Aurélie Dehaene, Rettinavelou Soundaravelou, Philippe Halfon, Anderson D. Loundou, Marie-Christine Alessi, Pierre-Emmanuel Morange

**Affiliations:** 1 Department of Critical Care, Hôpital Européen, Marseille, France; 2 C2VN, INSERM 1263, INRAE 1260, Aix Marseille University, Marseille, France; 3 Department of Hematology, Biogénopôle, Hôpital de la Timone, APHM, Marseille, France; 4 Department of Oncobiology, Hôpital Nord, APHM, Marseille, France; 5 Department of Radiology, Hôpital Européen, Marseille, France; 6 Department of Infectious Disease, Hôpital Européen, Marseille, France; 7 Department of Epidemiology, EA 3279-CEReSS, Aix Marseille University, Marseille, France; AIIMS: All India Institute of Medical Sciences, INDIA

## Abstract

**Background:**

Severe respiratory failure is a major complication of SARS-CoV-2 infection, and the need for mechanical ventilation (MV) is associated with a worse outcome. Whether some soluble biomarkers of lung injury can help predict MV requirement remains unclear.

**Methods:**

This prospective, observational, monocentric cohort study consecutively enrolled patients with laboratory-confirmed COVID-19 pneumonia within 48 h of hospital admission. The serum concentrations of five key bronchoalveolar epithelial and endothelial biomarkers were determined at Day 0, 7 and 14: Krebs von den Lungen-6 (KL-6); soluble receptor for advanced glycation end-products (sRAGE); club cell protein 16 (CC16); angiopoietin-2 (Ang-2); and soluble CD146 (sCD146). The respiratory severity of COVID-19 pneumonia was defined by the maximal level of respiratory support received during hospitalization: oxygen (by mask or nasal prong); high flow oxygen therapy (HFOT); and MV. End-points were the need for MV during hospitalization and the time to liberation from oxygen.

**Results:**

Fifty-four COVID-19 patients were enrolled; 23 (43%) required MV, 13 (24%) HFOT, and 18 (33%) oxygen. At inclusion, levels of KL-6, sRAGE, and CC16 were significantly higher in MV compared with non-MV patients (p < 0.05), with sRAGE showing the greatest difference (2.4-fold increase). In multivariate logistic regression, sRAGE (OR per 1000 pg/mL increase 1.316; 95% CI [1.040–1.667]; p = 0.022) and SpO_2_/F_I_O_2_ (OR 0.984; 95% CI [0.970–0.998]; p = 0.008) were identified as independent risk factors for MV. Furthermore, patients with an sRAGE ≥ 5449 pg/mL at inclusion had a lower probability of weaning from oxygen at Day 60 (HR 0.36; 95% CI [0.19–0.67]; p = 0.001). From Day 7 to Day 14, CC16 levels increased while sCD146 levels decreased in MV patients.

**Conclusion:**

Among five circulating biomarkers of bronchoalveolar injury, sRAGE showed the most favorable kinetic profile, rapidly increasing in MV patients. The early measurement of sRAGE and SpO_2_/F_I_O_2_ upon hospital admission may effectively identify COVID-19 patients at high risk of requiring MV and prolonged oxygen support.

## Introduction

Lung infection caused by the initial variants of severe acute respiratory syndrome coronavirus 2 (SARS-CoV-2), known as coronavirus disease 2019 (COVID-19), was most commonly associated with mild to moderate symptoms, but some patients developed severe hypoxemia and acute respiratory distress syndrome (ARDS) [1]. Predisposing risk factors for severe disease included older age, male sex, obesity, diabetes, hypertension, and chronic organ disease [2–5]. Among patients hospitalized with COVID-19 pneumonia, around 20% required admission to the intensive care unit (ICU) and those requiring mechanical ventilation (MV) had a worse outcome [3,6,7].

SARS-CoV-2 invades the bronchial epithelial cells, type I and type II alveolar pneumocytes, and capillary endothelial cells, ultimately causing cell death [8]. Monitoring circulating biomarkers, released by specific cell subtypes in response to damage, represents a noninvasive opportunity to quantify the extent of lung injury and predict the clinical severity of the disease [9].

The receptor for advanced glycation end-products (RAGE) is a transmembrane pattern recognition receptor constitutively expressed by type I pneumocytes [10]. The soluble form (sRAGE) is derived mainly from cleavage of the extracellular domain, and sRAGE levels indicate alveolar damage in ARDS [11,12]. In COVID-19 patients, sRAGE levels increase in relation to the clinical severity [13–17]. Krebs von den Lungen-6 (KL-6), a specific epitope of the high-molecular-weight glycosylated mucin 1 protein, is secreted by regenerating type II alveolar and bronchial epithelial cells to regulate fibroblast proliferation, migration, and survival [18,19]. KL-6 levels also increase in patients with severe COVID-19 pneumonia [20–27]. Club cell protein 16 (CC16) is a small secretory protein released by non-ciliated bronchial epithelial cells to mitigate the inflammatory response and facilitate epithelial regeneration [28–30]. A delayed increase in CC16 levels has been reported in severe COVID-19 cases [15,31].

Angiopoietin-2 (Ang-2) is an angiogenic factor released by endothelial cells upon activation [32]. COVID-19 patients with a delayed elevation of Ang-2 levels experience worse outcomes [15,33,34]. CD146 is an endothelial cell adhesion molecule involved in vessel integrity and cell migration [35,36]. Cleavage of the extracellular domain of the transmembrane glycoprotein liberates its soluble form (sCD146) [37]. One study reported higher plasma sCD146 levels in COVID-19 patients compared with healthy controls [38].

Although several studies have focused on circulating biomarkers of lung injury during COVID-19, none have compared these three prominent biomarkers of epithelial injury (i.e., sRAGE, KL-6, and CC-16), and the two biomarkers of endothelial injury (Ang-2 and sCD146). The aims of the current study were to describe the kinetics of these biomarkers in relation to disease severity and to determine whether these biomarkers could help predict the need for MV. Our hypothesis was that a combination of biomarkers with other parameters would reliably predict the requirement for MV. Some of the results of this study were presented in an abstract form at the *French Intensive Care Society International Congress* REANIMATION 2022 (FC-250) [39].

## Methods

This prospective, observational, cohort study was conducted at the European Hospital of Marseille, from 26^th^ November 2020 to 08^th^ June 2021, in accordance with the Declaration of Helsinki and the French law on research involving humans. The study protocol was approved by an independent national review board (Comité de Protection des Personnes, Ile de France XI, IDRCB 2020-A00756-33) and registered at ClinicalTrials.gov (NCT04816760). All patients signed written informed consent prior to inclusion. The reporting of this study follows the STROBE recommendations for cohort studies [40].

### Patient selection and management

Adult patients with symptomatic SARS-CoV-2 infection were consecutively screened for inclusion in the study if they fulfilled the following criteria: (i) hospitalized for ≤48 h; (ii) a positive SARS-CoV-2 reverse transcriptase-polymerase chain reaction test; (iii) evidence of COVID-19 pneumonia on a chest computed tomography (CT) scan (bilateral ground-glass opacities and/or consolidations); and (iv) acute onset of respiratory symptoms (≤1 week). Of note, according to local institutional protocols, oxygen supplementation was a prerequisite for hospital admission; thus, all included patients required oxygen therapy at the time of inclusion. The exclusion criteria were: pregnancy; immunosuppressive therapies within the previous 3 months (including cytotoxic chemotherapy); chronic respiratory failure with home oxygen or non-invasive ventilation; patients with a “do not resuscitate” order or an expected lifespan of <72 h; patients referred from another center with a length of stay >48 h; and patients unable to give informed consent.

Patients with COVID-19 pneumonia were managed according to the World Health Organization (WHO) living guideline [41], including standard anticoagulation in the absence of thrombosis, corticosteroid therapy (dexamethasone, 6 mg/day for 10 days) if requiring supplemental oxygen, and interleukin-6 receptor blocker therapy (tocilizumab, 8 mg/kg up to a maximum of 800 mg) in severe or critical illness. COVID-19 patients undergoing MV received low tidal volume protective ventilation, prone positioning if PaO_2_/F_I_O_2_ was ≤ 150 mmHg, and venovenous extracorporeal membrane oxygenation (ECMO) in accordance with guidelines [42]. Patients who developed clinical features of lung fibrosis defined by a worsening of respiratory system compliance (≤40 ml/cmH2O), persistent hypoxemia (PaO_2_/F_I_O_2_ ≤ 200), and CT features suggestive of lung fibroproliferation received rescue corticosteroid therapy (methylprednisolone, 2 mg/kg/day for 14 days) [43,44]. Twenty healthy individuals who regularly donated blood to the Etablissement Français du Sang served as contemporary controls to provide physiological baseline values for all biomarkers. These individuals were specifically selected to ensure the absence of chronic respiratory disease or systemic inflammation, representing a ‘gold standard’ for lung integrity.

### Samples and laboratory analysis

Blood samples were collected once from controls and at three time points (Day (D) 0, 7, and 14) from COVID-19 patients. The first sample (D0) was collected on the day of inclusion, which occurred within the first 48 h following hospital admission. The concentrations of five biomarkers of bronchoalveolar epithelial and endothelial lung injury were measured in the serum of controls and COVID-19 patients: KL-6; sRAGE; CC16; Ang-2; and sCD146. Whole blood samples were collected in SST™ II Advance BD Vacutainer® tubes, centrifuged for 10 min at 2300 × g, and serum was separated and immediately stored at −80°C for delayed analyses. Commercially available enzyme-linked immunosorbent assay (ELISA) kits from R&D Systems (Quantikine^®^, Minneapolis, MN, USA) were used to quantify sRAGE, CC16, and Ang-2, and from Biocytex (Cy-Quant^®^, Marseille, France) to quantify sCD146. All ELISA measurements were performed with the same lot number on thawed serum samples following recommendations from the manufacturer; the mean values of duplicated assays are reported. The accuracy of ELISA was assessed using replicates of the same control serum positioned at different locations within each plate. KL-6 levels were determined using a chemiluminescent enzyme immunoassay on a Lumipulse^®^ G1200 analyzer (Fujirebio, Tokyo, Japan). The others laboratory indices assessed in COVID-19 patients were complete blood count, neutrophil/lymphocyte ratio (NLR), creatinine, C-reactive protein (CRP), ferritin, lactate dehydrogenase (LDH), and D-dimer.

### Lung opacities assessment

Images from chest high resolution computed tomography (HRCT) were processed with *syngo*.via CT Pneumonia Analysis software (Version 1.0.4.2, Siemens Healthineers, Forchheim, Germany). This fully automated AI-based software delineates airspace opacities and provides lobe-wise opacity scores, percentage of opacity (relative to overall lung volume), and percentage of high opacity (relative to overall lung volume). To distinguish between ground glass opacities and consolidations, a Hounsfield Unit (HU) threshold of −200 HU was applied inside the detected airspace opacities. Areas denser than −200 HU were considered consolidations and classified as high opacities.

The opacity score was calculated according to the method reported by Bernheim et al. [45].

Each scan was evaluated for the mean total HU (whole lung parenchyma), opacity score (range: 0–20), percentage of opacity (range: 0–100%), and percentage of high opacity (range: 0–100%). Lung segmentations were controlled and manually adjusted if needed by two experienced radiologists. The performance of the algorithm has been reported elsewhere [46,47].

### Data collection and outcomes

The main demographic characteristics, comorbidities, medical history, length of hospital and ICU stays, types and duration of organ support, and hospital survival were recorded. The Simplified Acute Physiology Score II (SAPS II) was obtained at hospital admission [48], and the Sequential Organ Failure Assessment (SOFA) score was calculated on the days of measurements [49]. Respiratory severity was defined by the maximum type of respiratory support required during the hospital stay, according to the World Health Organization clinical progression scale (WHO-CPS) [50]: oxygen by mask or nasal prong [WHO-CPS grade 5]; high flow oxygen therapy (HFOT) [WHO-CPS grade 6]; and MV [WHO-CPS grade 7–9]. Oxygen-free days were defined as the number of days alive and free from oxygen therapy between inclusion and the time point of interest. The primary outcome was the requirement for MV during the hospital stay. Secondary outcomes included weaning from oxygen and oxygen-free days at D28 and D60, and hospital survival.

### Statistical analysis

The required sample size was determined based on the objective of estimating the proportion of patients requiring mechanical ventilation (MV) with a pre-specified precision (S1 Appendix). Fifty-four subjects were needed to estimate the true population proportion. Twelve missing data (1.2%), most likely missing at random, and involving five variables (four from ferritin, two from LDH, two from D-dimer, two from % of opacity, and two from % of high opacity) were imputed using the multivariate imputation by chained equation (mice) R statistical package (R 4.4.1, R Core Team, 2024) [51].

Categorical data are presented as number and percentage (%), and were compared using the Chi-square test or Fisher’s exact test. Continuous data are presented as median and interquartile range [IQR 25–75%], and were compared using nonparametric tests. Comparisons between controls and COVID-19 subgroups were performed using the Kruskal-Wallis test followed by Conover’s test for *post-hoc* multiple comparisons. Data for COVID-19 patients stratified by MV were compared using the Mann-Whitney U test and Odds ratios (ORs) with 95% confidence intervals [95% CI] were determined by univariate logistic regression. The performance of the biomarkers to predict MV and weaning from oxygen was assessed by the area under curve (AUC) through receiver operating characteristic (ROC) analysis, and the criterion was determined by the Youden index method. Risk factors for MV were determined by multivariate stepwise logistic regression and risk factors for weaning from oxygen were determined using Cox proportional-hazard regression. The time to weaning from oxygen was compared using the log rank test and was represented with Kaplan-Meier curves. The correlations between variables were assessed using Spearman’s coefficient of rank correlation (ρ). Biomarker kinetics were investigated using generalized linear model analyses (GLM) to test the effect of time (D0, 7, and 14), MV (binary), and their interaction. All tests were two-tailed and the significance level was set at 5%.

Statistical analyses were carried out using IBM SPSS Statistics (v29.0.2.0 Armonk, NY, USA) and graphics were created using MedCalc Statistical Software (v23.0.2, Ostend, Belgium) and the ggplot2 R package [52].

## Results

### Study population

Fifty-four patients with COVID-19 pneumonia and 20 healthy controls were included in the study. The main baseline characteristics are reported in Table 1. The flow chart of the study population is shown in S1 Fig in the Appendix. Eighteen COVID-19 patients (33%) were managed on the ward and received only oxygen therapy during hospitalization. Thirty-six COVID-19 patients (67%) required ICU admission; of these 13 (24%) received HFOT and 23 (43%) required MV. Among the 23 MV patients, 21 (91%) received prone positioning, four (17%) required venovenous ECMO, 18 (78%) received norepinephrine, and five (9%) needed renal replacement therapy.

**Table 1:** Baseline characteristics of control subjects and COVID-19 patients.

Variables, units	Controls	COVID-19			
		All	Oxygen	HFOT	MV
No. of subjects	20	54	18	13	23
Age, yrs	44 [34–53]	63 [53–71]	60 [53–72]	56 [48–69]	66 [57 –71]
Male, n (%)	14 (70)	41 (76)	12 (67)	10 (77)	19 (83)
BMI, kg/m²	25 [23 –27]	29 [26 –31]	27 [24 –31]	28 [26 –29]	31 [29 –34]
**Comorbidities,** n (%)					
Diabetes	0	20 (37)	8 (44)	4 (31)	8 (35)
Hypertension	0	23 (43)	5 (28)	5 (38)	13 (57)
Chronic pulmonary diseases	0	6 (11)	2 (11)	3 (23)	1 (4)
Chronic heart diseases	0	8 (15)	3 (17)	1 (8)	4 (17)
Chronic kidney diseases	0	0	–	–	–
Chronic liver diseases	0	0	–	–	–
Active cancer	0	2 (4)	1 (6)	1 (8)	0
Immunosuppression	0	0	–	–	–
COVID-19 vaccine prior inclusion, n (%)	–	2 (4)	1 (6)	0	1 (4)
Antiviral agent prior inclusion, n (%)	–	1 (2)	0	1 (8)	0
Time from first symptom to hospital, days	–	7 [5 –9]	7 [4 –9]	8 [7 –10]	7 [5 –8]
Time from first symptom to inclusion, days	–	9 [6 –11]	7 [5 –11]	9 [8 –11]	9 [7 –11]
SAPS II, at hospital admission	–	24 [18 –34]	18 [17 –21]	24 [17 –33]	33 [23–42]
SOFA score, at inclusion	–	2 [2 –3]	2 [1 –2]	2 [2 –2]	4 [2 –8]
SpO_2_/F_I_O_2_, at inclusion	–	187 [133–300]	341 [297–452]	160 [144–233]	130 [99–185]
**Immunomodulatory drugs,** n (%)					
Early dexamethasone therapy	–	50 (93)	14 (78)	13 (100)	23 (100)
Dexamethasone prior inclusion	–	37 (69)	10 (56)	10 (77)	17 (74)
Rescue methylprednisolone therapy	–	13 (24)	0	0	13 (57)
IL-6 receptor blocker therapy	–	7 (13)	0	2 (15)	5 (22)
IL-6 receptor blocker prior to inclusion	–	2 (4)	0	1 (8)	1 (4)
**Outcomes**					
Length of hospital stay, days	–	13 [6–32]	6 [4 –8]	10 [8 –13]	39 [27–61]
Length of MV, days	–	–	–	–	16 [8–32]
Oxygen-free days at Day 28, days	–	18 [0–23]	25 [23 –26]	20 [16 –22]	0 [0–0]
Oxygen-free days at Day 60, days	–	50 [24–55]	57 [55 –58]	52 [48 –54]	11 [0–29]
28-day mortality, n (%)		3 (6)	0	0	3 (6)
In-hospital mortality, n (%)	–	7 (13)	0	0	7 (30)

*Definition of abbreviations*: HFOT = high flow oxygen therapy; MV = mechanical ventilation; BMI = body mass index; SAPS = Simplified Acute Physiology Score; SOFA = Sequential Organ Failure Assessment.

COVID-19 subgroups were defined according to the maximal level of respiratory support received during hospitalization on the World Health Organization clinical progression scale (WHO-CPS). Data are expressed as number (%) or median [interquartile range 25–75%].

### Lung biomarker profiles across respiratory severity groups

**Fig 1:**
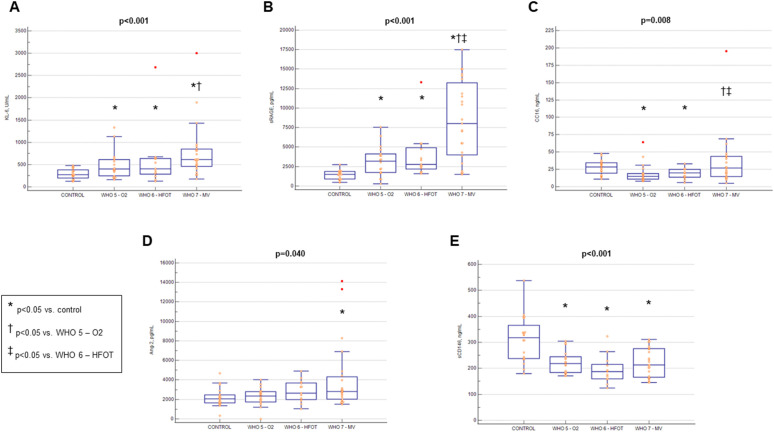
Serum levels of bronchoalveolar epithelial and endothelial biomarkers at inclusion. Serum samples were obtained within the first 48 h of hospital admission from 54 COVID-19 patients and 20 healthy controls. COVID-19 subgroups were defined according to the maximal level of respiratory support received during hospitalization on the World Health Organization clinical progression scale (WHO-CPS): oxygen by mask or nasal prong (O_2_), high flow oxygen therapy (HFOT), and mechanical ventilation (MV). **(A)** Krebs von den Lungen-6 (KL-6). **(B)** soluble receptor for advanced glycation end-products (sRAGE). **(C)** Club cell protein 16 (CC16). **(D)** Angiopoietin-2 (Ang-2). **(E)** soluble CD146 (sCD146). Statistical analyses were performed using the Kruskal-Wallis test and *post-hoc* multiple comparisons with the Conover test..

The median delay between hospital admission and the first blood sample (D0) was 1 [1; 2] day without differences between COVID-19 subgroups (S1 Table). The serum levels of bronchoalveolar epithelial and endothelial biomarkers are shown in Fig 1 and values are reported in S2 Table. KL-6 levels were higher in the oxygen, HFOT, and MV groups compared with controls, and were higher in the MV group compared with the oxygen group. sRAGE levels were higher in all COVID-19 subgroups compared with controls, and were higher in the MV group compared with the oxygen and HFOT groups. CC16 levels were lower in the oxygen and HFOT groups than in controls and the MV group. Ang-2 levels were higher in the MV group compared with controls. sCD146 levels were lower in all COVID-19 subgroups compared with controls, with no differences observed among COVID-19 subgroups.

The results of laboratory indices and CT-related parameters among COVID-19 subgroups are presented in S3 Table. Lung CT scans were performed either prior to (n = 52) or after (n = 2) inclusion, and the median delay between the CT scan and the first blood sample was −2 [−2;-1] days. The quantity of opacities gradually increased with respiratory severity whilst the mean Hounsfield Unit (HU) of the whole lung parenchyma decreased.

### Lung Biomarkers According to Mechanical Ventilation Requirement

**Fig 2:**
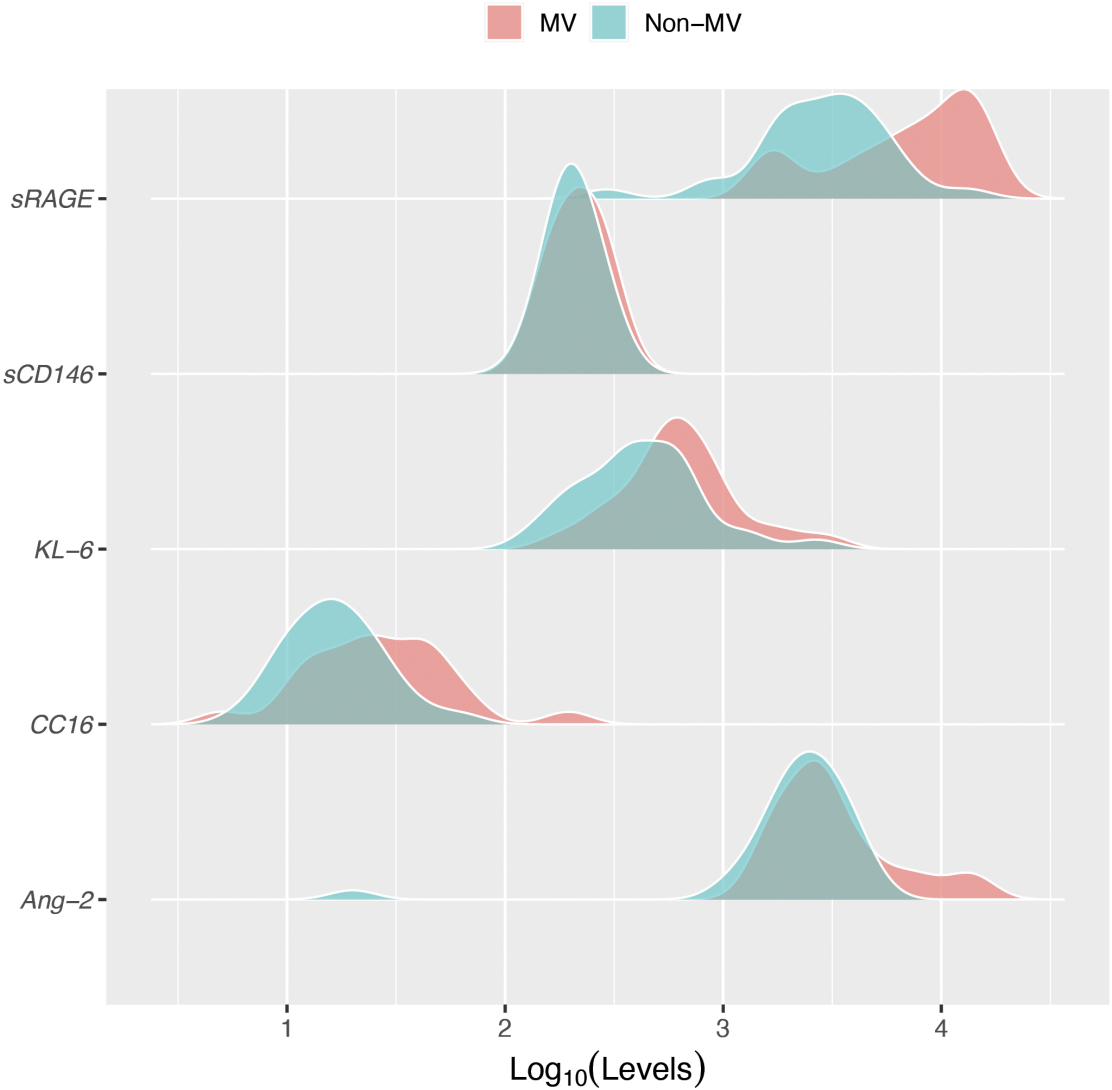
Density distribution of bronchoalveolar biomarkers in COVID-19 patients stratified by mechanical ventilation. Serum samples were obtained within the first 48 h of hospital admission from 54 COVID-19 patients. The density distribution of five bronchoalveolar epithelial and endothelial biomarkers is represented: Krebs von den Lungen-6 (KL-6), soluble receptor for advanced glycation end-products (sRAGE), club cell protein 16 (CC16), Angiopoietin-2 (Ang-2), and soluble CD146 (sCD146). The distribution of COVID-19 patients who required mechanical ventilation (MV) is colored in red, while those who did not require MV (non-MV) are shown in green. The horizontal axis represents the Log_10_ levels of biomarkers measured in pg/mL for sRAGE and Ang-2, in ng/mL for CC16 and sCD146, and in U/mL for KL-6.

Among the 23 COVID-19 patients requiring MV, the median delay between MV initiation and the first blood sample was 0 [−1; 2] day. Seven patients received MV prior to inclusion within a median delay of −1 [−2;-1] day. The main characteristics of the COVID-19 patients stratified by MV are presented in S4 Table. MV patients had higher body mass index (BMI), SOFA score, and lower SpO_2_/F_I_O_2_ ratio at inclusion. Laboratory indices (NLR, D-dimer, CRP, ferritin, LDH, and creatinine) and CT-related parameters (% of opacity and % of high opacity) were also higher in MV patients. Among the bronchoalveolar biomarkers, only KL-6, sRAGE, and CC16 levels were increased in MV patients (Fig 2). Four variables (NLR, SOFA score, sRAGE, and % of high opacity) exhibited a ≥ 2-fold increase. Five variables (CRP, CC16, ferritin, Ang-2, and D-dimer) exhibited a 1.5- to 2-fold increase, and one variable (SpO_2_/F_I_O_2_) decreased by 0.5-fold (S2 Fig).

The predictive performance of biomarkers to predict the requirement for MV is reported in S5 Table. The highest AUC was achieved by the SpO_2_/F_I_O_2_ ratio (AUC 0.861; 95% CI [0.740–0.940]; p < 0.001) followed by NLR (AUC 0.836; 95% CI [0.710–0.923]; p < 0.001). Among the biomarkers, the highest AUC was achieved by sRAGE (AUC 0.786; 95% CI [0.653–0.886]; p < 0.001), with a sensitivity of 69.6% and a specificity of 90.3%. In a sensitivity analysis excluding the seven patients receiving MV prior to inclusion, the AUC of sRAGE to predict MV was 0.888 (95% CI [0.762–0.961]; p < 0.001), with a sensitivity of 81.2% and a specificity of 90.3%.

The ORs and 95% CI for MV by univariate and multivariate logistic regression are reported in Table 2. After adjusting for confounders, the independent risk factors for MV were sRAGE (OR per 1000 pg/mL increase 1.316; 95% CI [1.040–1.667]; p = 0.022) and SpO_2_/F_I_O_2_ ratio (OR 0.984; 95% CI [0.970–0.998]; p = 0.008). The model correctly classified 79.6% of cases (AUC 0.899; 95% CI [0.786–0.964]; p < 0.001).

**Table 2:** Risk factors for mechanical ventilation in COVID-19 patients.

Variables, units	Unadjusted Odds Ratio	95% CI	p value	Adjusted Odds Ratio	95% CI	p value
Age, yrs	1.038	0.990–1.088	0.120	–	–	–
Male	0.515	0.136–1.942	0.327	–	–	–
BMI, kg/m²	1.188	1.034–1364	**0.015**	–	–	–
SOFA score	4.213	1.488–11.929	**0.007**	–	–	–
SpO_2_/F_I_O_2_	0.982	0.972–0.992	**<0.001**	0.985	0.974–0.996	**0.008**
Percentage of opacity	1.052	1.021–1.083	**0.001**	–	–	–
Percentage of high opacity	1.102	1.029–1.179	**0.006**	–	–	–
Neutrophil/lymphocyte ratio	1.199	1.065–1.350	**0.003**	–	–	–
D-dimer, µg/mL	1.233	0.931–1.633	0.143	–	–	–
C-reactive protein, mg/L	1.011	1.004–1.019	**0.004**	–	–	–
Ferritin, µg/L	1.001	1.000–1.002	0.062	–	–	–
Lactate dehydrogenase, U/L	1.008	1.004–1.013	**0.001**	–	–	–
Creatinine, µmol/L	1.024	1.000–1.050	0.052	–	–	–
Krebs von den Lungen-6, U/mL	1.001	1.000–1.002	0.138	–	–	–
sRAGE, pg/mL (per 1000 pg/mL increase)	1.395	1.145–1.699	**0.001**	1.316	1.040–1.667	**0.022**
Club cell protein 16, ng/mL	1.051	1.006–1.098	**0.026**	–	–	–
Angiopietin-2, pg/mL	1.000	1.000–1.001	0.063	–	–	–
sCD146, ng/mL	1.004	0.994–1.015	0.417	–	–	–

*Definition of abbreviations*: CI = confidence interval; BMI = body mass index; SOFA = Sequential Organ Failure Assessment; sRAGE = soluble receptor for advanced glycation end-products; sCD146 = soluble CD146.

Data were obtained within the first 48 h of hospital admission in 54 COVID patients. Unadjusted Odds Ratios (ORs) were determined by univariate logistic regression and adjusted ORs by multivariate stepwise logistic regression. Boldface type indicates statistical significance.

### Correlations between lung biomarkers and clinical parameters

**Fig 3:**
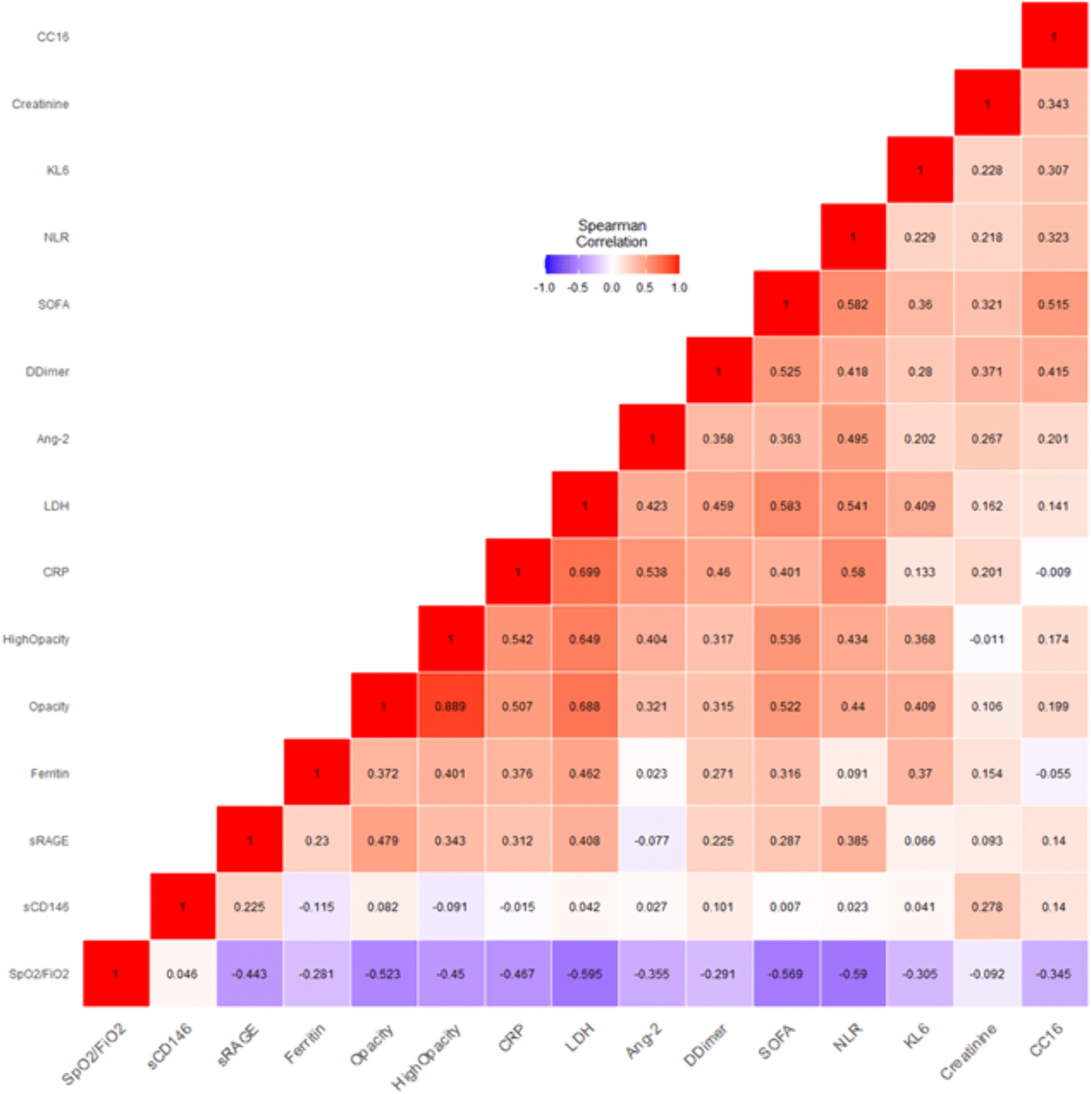
Heat map illustrating biomarker correlations at inclusion in COVID-19 patients. Measurements were performed within the first 48 h of hospital admission in 54 COVID-19 patients. Correlations were determined between biomarkers of bronchoalveolar epithelial and endothelial injury (Krebs von den Lungen-6 (KL-6), soluble receptor for advanced glycation end-products (sRAGE), Club cell protein 16 (CC16), Angiopoietin-2 (Ang-2), and soluble CD146 (sCD146)), laboratory indices (creatinine, C-reactive protein (CRP), lactate dehydrogenase (LDH), ferritin, D-dimer, and neutrophil/lymphocyte ratio (NLR)), CT-related parameters (percentage of opacity, and percentage of high opacity), respiratory severity index (ratio of peripheral oxygen saturation to inspired oxygen fraction (SpO_2_/F_I_O_2_)), and severity score (Sequential Organ Failure Assessment (SOFA)). Spearman’s rank correlation coefficients are reported.

The whole set of correlations is summarized through a heat map in Fig 3. The biomarkers were statistically associated with several variables. Notably, the percentage of opacity correlated with the levels of sRAGE (ρ = 0.475; p < 0.001), KL-6 (ρ = 0.390; p = 0.004), and Ang-2 (ρ = 0.307; p = 0.027). The percentage of high opacity correlated with Ang-2 (ρ = 0.4; p = 0.003), KL-6 (ρ = 0.358; p = 0.009), and sRAGE (ρ = 0.356; p = 0.009). Negative correlations were observed between the SpO_2_/F_I_O_2_ ratio and the levels of sRAGE (ρ = −0.443; p < 0.001), Ang2 (ρ = −0.355; p < 0.001), CC16 (ρ = −0.345; p = 0.011), and KL-6 (ρ = −0.305; p = 0.025). Finally, oxygen-free days at D60 negatively correlated with the levels of CC16 (ρ = −0.448; p < 0.001), sRAGE (ρ = −0.413; p = 0.002), Ang-2 (ρ = −0.362; p = 0.007), and KL-6 (ρ = −0.267; p = 0.05).

### Lung biomarker kinetics stratified by mechanical ventilation

**Fig 4:**
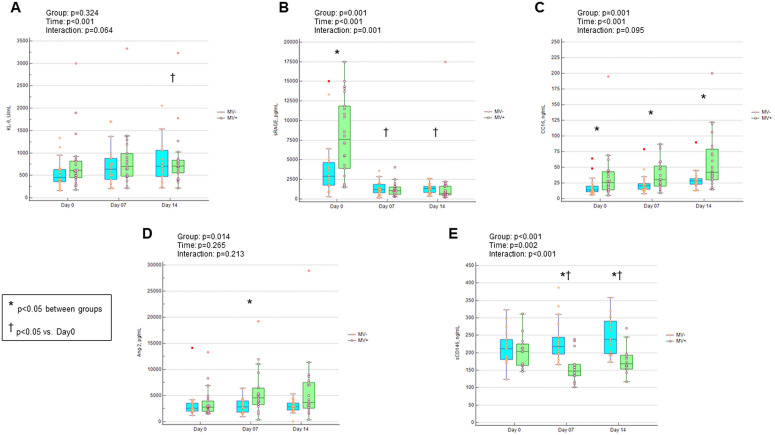
Kinetics of bronchoalveolar epithelial and endothelial biomarkers over 14 days in COVID-19 patients. Serum samples were obtained from 44 COVID-19 patients at inclusion (Day 0 (D0)) and at D7 and D14 following inclusion. COVID-19 patients were stratified by the need for MV during hospitalization into two groups (MV+ and MV-), both including 22 patients. **(A)** Krebs von den Lungen-6 (KL-6). **(B)** soluble receptor for advanced glycation end-products (sRAGE). **(C)** Club cell protein16 (CC16). **(D)** Angiopoietin-2 (Ang-2). **(E)** soluble CD146 (sCD146). Statistical analyses were performed with generalized linear models (GLM) to test the effect of group (MV), time (day of measurement), and interaction.

Complete biomarker kinetics (D0, 7, and 14) were available for 44 COVID-19 patients, among which 22 (50%) received MV (S6 Table). The kinetics of KL-6 were time dependent, KL-6 levels being higher at D14 compared with D0 (Fig 4A). The kinetics of sRAGE were group and time dependent. sRAGE levels peaked at D0 and were higher in MV patients than in non-MV patients. At D7 and D14, sRAGE levels had decreased compared with D0 and were not different between MV and non-MV patients (Fig 4B). The kinetics of CC16 were group and time dependent. CC16 levels increased gradually at each time point in the MV group compared with the non-MV group (Fig 4C). The kinetics of Ang-2 were group dependent; Ang-2 levels were higher at D7 in the MV group (Fig 4D). The kinetics of sCD146 were group and time dependent; sCD146 levels decreased at D7 and D14 in MV patients (Fig 4E).

### Lung biomarkers and weaning from oxygen

At Day 28, 36 COVID-19 patients (67%) were alive and weaned from oxygen whilst 46 (85%) achieved weaning and seven (13%) had died at D60. At inclusion, sRAGE achieved the highest AUC to predict weaning from oxygen at D28 (AUC 0.796; 95%CI [0.653–0.886]; p < 0.001), while CC16 showed the best performance for weaning at D60 (AUC 0.738; 95%CI [0.600–0.848]; p = 0.008) (S7 and S8 Tables).

**Fig 5:**
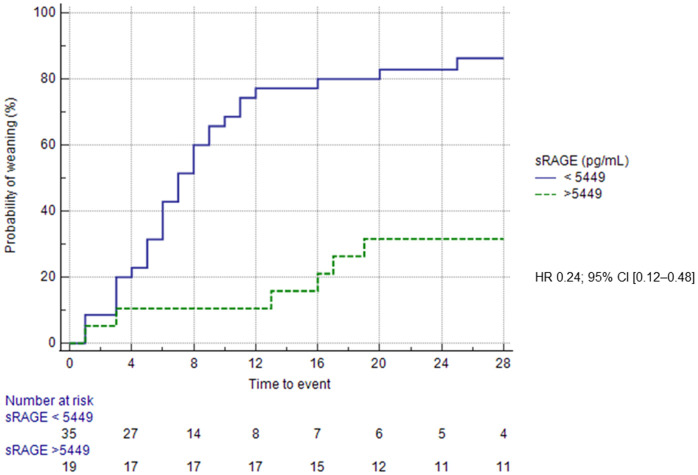
Probability of weaning from oxygen in COVID-19 patients stratified by the sRAGE level at inclusion. The soluble receptor for advanced glycation end-products (sRAGE) levels were quantified in the serum of 54 COVID-19 patients within the first 48 h of hospital admission. The sRAGE threshold (5449 pg/mL) was determined by the Youden index method. The cumulative probability of weaning from oxygen until Day 28 is reported on the y-axis. Deceased patients (n = 7) were censored. Statistical analysis was performed with the Log-rank test to estimate the Hazard ratio (HR) and the 95% confidence interval (CI). Patients with sRAGE levels ≥ 5449 pg/mL had a lower probability of weaning..

In Cox proportional-hazard regression, four independent factors were associated with the risk of weaning from oxygen at D28: sRAGE (HR per 1000 pg/mL increase 0.900; 95% CI [0.811–0.999]; p = 0.050), SpO_2_/F_I_O_2_ (HR 1.007; 95% CI [1.004–1.010]; p < 0.001), age (HR 0.964; 95% CI [0.938–0.990]; p = 0.007), and BMI (HR 0.881; 95% CI [0.810–0.957]; p = 0.003). Based on the optimal threshold determined by the Youden Index (≥5449 pg/mL; sensitivity 83.3%, specificity 72.2%), COVID-19 patients with sRAGE levels above this cut-off at inclusion had a lower probability of weaning from oxygen at D28 (HR 0.24; 95% CI [0.12–0.48]; p < 0.001, Fig 5) and D60 (HR 0.36; 95% CI [0.19–0.67]; p = 0.001).

## Discussion

This study shows that in hospitalized patients with moderate to severe COVID-19 pneumonia sRAGE had the most favorable kinetic profile among the biomarkers studied with a 2.4-fold increase in MV patients at hospital admission whereas KL-6 was associated with poor discrimination and CC16, Ang-2, and sCD146 had delayed alterations. sRAGE measured within the first 48 h of hospital admission was an independent risk factor for MV and was associated with a lower probability of weaning from oxygen up to D60.

During the course of severe COVID-19 pneumonia, some studies have suggested that circulating biomarkers originating from the alveolar epithelium are released before those from the capillary endothelium [15,53]. Leisman et al. reported a 1.8-fold increase in RAGE levels at admission in the plasma of intubated patients whilst endothelial injury (Ang-2) and Club cell (CC16) biomarkers increased by D3 [15]. The results of our study corroborate and extend these findings. First, we observed a 2.4-fold increase in sRAGE levels at admission in the serum of MV patients that resolved by D7. Second, we confirmed the delayed elevation of Ang-2 and CC16 in MV patients, by D7 in our study. Finally, our results add new findings at D14 showing that CC16 levels continued to increase in MV patients whilst Ang-2 levels decreased. Thus, the kinetic profiles of these biomarkers provide insights into the severity and stage of the disease.

The present study is the first to compare the performance of KL-6 with sRAGE and CC16 in COVID-19 patients. In line with previous reports [20–27], higher KL-6 levels were observed at admission in MV patients, but the discriminating performance of KL-6 was weaker than that of sRAGE. KL-6 levels tended to increase over time without any difference between MV and non-MV patients. Surprisingly, both KL-6 and sRAGE were unaffected by MV status at D7 and D14, in contrast to CC16. Conversely, CC16 levels at hospital admission were poorly discriminant as patients with moderate to severe disease exhibited lower levels than healthy controls. While this paradoxical initial decrease has been reported previously [54,55], our study uniquely shows a progressive and marked increase in CC16 levels up to D14 specifically in patients under MV. This sustained elevation of CC16, occurring while sRAGE levels have already normalized, suggests that CC16 might track different pathological processes, such as persistent bronchoalveolar barrier disruption or secondary injury associated with prolonged mechanical ventilation [54,56]. Our data suggest that these biomarkers provide complementary rather than redundant temporal information regarding the evolution of lung injury in COVID-19.

Our study provides original insights into sCD146, an emerging biomarker of endothelial activation that remains sparsely investigated in COVID-19. While the only available report to date by Syed et al. [38] showed increased plasma sCD146 levels, we observed a significant decrease in serum levels at admission, particularly in patients requiring MV. Despite extensive literature searches, sCD146 remains a rare target in COVID-19 research, but our findings of decreased serum levels are consistent with patterns observed in other acute inflammatory states [57,58]. The biological significance of this decrease during the acute phase remains to be fully elucidated. Several non-exclusive mechanisms could be hypothesized: (i) a downregulation of the CD146 receptor expression on the endothelial surface, (ii) a reduction in the proteolytic cleavage (shedding) of its extramembranous part, or (iii) an accelerated intravascular degradation of the soluble form by circulating enzymes. Although our study was not designed to explore these mechanistic pathways, it is noteworthy that sCD146 and Ang-2 exhibited mirrored kinetics over time. In patients requiring MV, both biomarkers showed a gradual and synchronized variation from admission through D14, albeit in opposite directions. This suggests that while their biological regulation differs—likely involving increased release for Ang-2 versus potential downregulation or consumption for sCD146—both markers consistently and concertedly reflect the progression of endothelial involvement in the most severe cases.

Determining the strength of the relation between a biomarker and the extent of lung injury yields valuable information to compare biomarkers [59]. In this study, AI-based software was used to quantify lung opacities, showing that both the percentage of opacity and the mean HU (Hounsfield Units) increased gradually with the level of respiratory support, indicating a progressive loss of lung aeration. LDH achieved the highest level of correlation with both the percentage of radiological opacity and the severity of hypoxemia. With a slightly lower strength of correlation than LDH, sRAGE was the lung-specific biomarker that exhibited the highest level of correlation with these two parameters. However, when comparing their performance for predicting the need for MV, sRAGE (AUC 0.786, specificity 90.3%) proved to be a more specific indicator than LDH (AUC 0.810, specificity 83.9%). Indeed, only sRAGE and SpO_2_/F_I_O_2_ remained independently associated with the risk of MV in multivariate analysis (Table 2). This suggests that while LDH reflects global lung tissue damage, sRAGE more specifically tracks the alveolar epithelial insult that drives progressive respiratory failure.

While risk factors for MV such as age, comorbidities, and standard laboratory markers are well-documented [60–62], few studies have evaluated the independent weight of lung-specific injury biomarkers in models integrating clinical, radiological, and severity scores. In this comprehensive context, sRAGE and the SpO_2_/F_I_O_2_ ratio remained independent predictors of MV in our cohort. Notably, each 1000 pg/mL increase in sRAGE at admission was associated with a 32% increase in the risk of requiring MV. These results extend the findings of Lim et al. [14] by demonstrating that alveolar epithelial damage is a primary driver of respiratory failure, independent of systemic inflammation. Furthermore, the prognostic value of sRAGE extends beyond the acute phase, as admission levels were associated with a lower probability of oxygen weaning at D60. This suggests that the initial magnitude of the epithelial insult dictates not only the immediate need for life support but also the trajectory of lung recovery.

The biomarker profiles observed in our study share similarities with those found in non-COVID-19 ARDS, though some specificities exist. sRAGE is a recognized hallmark of alveolar epithelial type I cell injury [11,12]. Interestingly, previous studies have shown that sRAGE levels are significantly higher in patients with COVID-19-related ARDS than in those with ARDS from other etiologies [17,53]. This may be explained by specific molecular interactions, as the SARS-CoV-2 nucleocapsid (N) protein has recently been identified as a high-affinity ligand for RAGE, potentially magnifying the epithelial injury signal [63]. Furthermore, circulating levels of the SARS-CoV-2 N-antigen have been positively correlated with sRAGE concentrations, suggesting that systemic viral load is directly linked to the extent of alveolar epithelial insult [64]. In contrast, markers of endothelial activation (Ang-2) or distal airway injury (CC16) followed patterns in our cohort more similar to those described in classical ARDS, where their elevation is often related to the progression of organ support rather than the initial viral-induced epithelial hit.

This study has some limitations. First, most COVID-19 patients (93%) received corticosteroids during their hospital stay, even prior to inclusion in 69% of cases. While these treatments significantly decrease systemic inflammatory markers [65], their impact on lung injury biomarkers remains poorly documented with contradictory findings. In a pre-specified analysis of a multicentric cohort, Angiopoietin-2 levels were not significantly altered by corticosteroid therapy in COVID-19 [66]. Although another small subgroup analysis suggested a faster decline in sRAGE and Ang-2 under dexamethasone [67], this finding was limited by a major imbalance in disease severity between groups. To our knowledge, no specific data are currently available regarding the impact of corticosteroids on other lung injury biomarkers such as CC16, KL-6, or sCD146 in this context. Since the proportion of patients receiving corticosteroids prior to inclusion was similar between our MV and non-MV groups, it is unlikely that these treatments biased our results at admission. Second, MV was initiated prior to the first blood sample in seven of the 23 patients requiring MV. While this could theoretically influence biomarker levels, a sensitivity analysis excluding these seven patients actually yielded a higher AUC for sRAGE (0.888 vs. 0.786) and improved sensitivity (81.2% vs. 69.6%), with an identical optimal cut-off (5449 pg/mL). This suggests that sRAGE levels primarily reflect the severity of the underlying alveolar insult rather than the impact of MV itself. However, we acknowledge that for strict predictive modeling, future studies should ensure that all samples are collected prior to any escalation in respiratory support level. Third, the study was conducted in a single center with a limited sample size, which resulted in relatively narrow confidence intervals near the unit for some predictors. While statistically significant, these findings should be interpreted with caution and require validation in larger, multicentric cohorts. Fourth, our study’s observational design led us to report Odds Ratios (OR) rather than Relative Risks (RR). Because the incidence of the primary endpoint (MV) was relatively high in our cohort (43%), the OR may overestimate the RR and should be interpreted as a measure of association rather than a direct risk ratio. Fifth, although we identified sRAGE as a strong independent predictor, its ultimate clinical relevance—particularly for real-time decision-making—remains to be established through prospective trials comparing outcomes in patients stratified by sRAGE levels at admission. Finally, our findings should be interpreted within the context of the first SARS-CoV-2 variants and the standard use of dexamethasone, and their generalizability to more recent viral strains or different therapeutic regimens remains to be confirmed.

## Conclusion

In conclusion, this study identifies sRAGE levels at admission and the SpO_2_/F_I_O_2_ ratio as independent predictors of the need for mechanical ventilation in patients hospitalized for COVID-19 pneumonia. A key finding is the early and transient elevation of sRAGE, which distinguishes it from other lung injury biomarkers that showed delayed or less discriminative alterations. While sRAGE demonstrates a robust association with the need for ventilatory support (OR 1.32 per 1000 pg/mL increment), these findings should be interpreted within the context of a single-center study with a limited sample size and the specific era of early SARS-CoV-2 variants and dexamethasone therapy. Furthermore, given the high incidence of the primary endpoint, the use of Odds Ratios rather than Relative Risks may overestimate the association. Ultimately, the clinical relevance of sRAGE for real-time decision-making remains to be established through larger, multicentric prospective trials.

## Supporting information

S1 Appendix Sample size determination. (PDF)

S1 Fig Flow chart of the study population. Definition of abbreviations: ICU = intensive care unit; O2 = standard oxygen therapy (nasal prong or face mask); HFOT = high flow oxygen therapy; MV = mechanical ventilation.
(TIF)

S2 Fig Volcano plot showing fold changes in baseline parameters of COVID-19 patients requiring mechanical ventilation. Measurements in COVID-19 patients were performed within the first 48 h of hospital admission. COVID-19 patients were stratified by the need for mechanical ventilation (MV) during hospitalization into two groups. Fold changes (FC) are represented on a log2 scale in the x-axis. False Discovery Rate (FDR) p values are represented on a log10 scale in the y-axis. Labelled variables are those with fold changes <0.5 or >1.5 and FDR p values ≤0.05. SpO_2_/F_I_O_2_ = peripheral oxygen saturation to inspired oxygen fraction ratio; SOFA = Sequential Organ Failure Assessment; NLR = neutrophil/lymphocyte ratio; sRAGE = soluble receptor of advanced glycation end-products; CC16 = Club cell protein 16.
(TIF)

S1 Table Time interval between hospital admission and Day 0 blood sampling according to respiratory support. Definition of abbreviations: HFOT = high flow oxygen therapy; MV = mechanical ventilation. COVID-19 subgroups were defined according to the maximal level of respiratory support received during hospitalization on the World Health Organization clinical progression scale (WHO-CPS). Data are expressed as number (%). Statistics were performed with Chi-2 test or Fisher’s exact test.
(PDF)

S2 Table Serum levels of lung epithelial and endothelial biomarkers at inclusion in healthy controls and COVID-19 patients according to respiratory support. Definition of abbreviations: HFOT = high flow oxygen therapy; MV = mechanical ventilation; sRAGE = soluble receptor of advanced glycation end-products; sCD146 = soluble CD146. Data are presented as median [interquartile range: 25–75%]. Measurements in COVID-19 patients were performed within the first 48 h of hospital admission. Statistical analyses were performed with the Kruskal-Wallis test and post-hoc multiple comparisons with the Conover test. Boldface type indicates statistical significance. * p < 0.05 vs. controls; † p < 0.05 vs. oxygen group; ‡ p < 0.05 vs. HFOT group.
(PDF)

S3 Table Baseline laboratory indices and CT-related parameters in COVID-19 patients according to respiratory support. Definition of abbreviations: HFOT = high flow oxygen therapy; MV = mechanical ventilation; HU = Hounsfield unit. COVID-19 subgroups were defined according to the maximal level of respiratory support received during hospitalization on the World Health Organization clinical progression scale (WHO-CPS). Data are presented as median [interquartile range: 25–75%]. Statistical analyses were performed with the Kruskal-Wallis test and post-hoc multiple comparisons with the Conover test for continuous variables. Boldface type indicates statistical significance. * p < 0.05 vs. oxygen group; † p < 0.05 vs. HFOT group.
(PDF)

S4 Table Baseline characteristics of COVID-19 patients stratified by the need for mechanical ventilation during hospitalization. Definition of abbreviations: BMI = body mass index; SOFA = Sequential Organ Failure Assessment; sRAGE = soluble receptor for advanced glycation end-products; sCD146 = soluble CD146. Data are expressed as number (percentage) or median [interquartile range: 25–75%]. Statistical analyses were performed with the Mann-Whitney U test for continuous variables and the Chi-square test or Fisher’s exact test for categorical variables. Boldface type indicates statistical significance.
(PDF)

S5 Table Receiver operating characteristics analyses of lung biomarkers and clinical parameters at inclusion to predict the need for mechanical ventilation during hospitalization. Definition of abbreviations: AUC = area under the curve; CI = confidence interval; Se = sensitivity; Sp = specificity; KL-6 = Krebs von den Lungen-6; sRAGE = soluble receptor of advanced glycation end-products; CC16 = Club cell protein 16; Ang-2 = Angiopoietin-2; sCD146 = soluble CD146; LDH = lactate dehydrogenase; BMI = body mass index; SOFA = Sequential Organ Failure Assessment. Measurements were performed within the first 48 h of hospital admission in 54 COVID-19 patients. The criterion was determined by the Youden index method. Boldface type indicates statistical significance.
(PDF)

S6 Table Kinetics of lung epithelial and endothelial biomarkers in COVID-19 patients stratified by the need for mechanical ventilation during hospitalization. Definition of abbreviations: MV = mechanical ventilation; KL-6 = Krebs von den Lungen-6; sRAGE = soluble receptor of advanced glycation end-products; CC16 = Club cell protein 16; Ang-2 = Angiopoietin-2; sCD146 = soluble CD146. Data are presented as median [interquartile range: 25–75%]. Measurements were performed in 44 COVID-19 patients at inclusion (Day 0) and at Day 7 and Day 14 following inclusion. COVID-19 patients were stratified by the need for MV during hospitalization into MV (N = 22) and Non-MV (N = 22) groups. Statistical analyses were performed with the Kruskal-Wallis test and post-hoc multiple comparisons with the Conover test. Boldface type indicates statistical significance. * p < 0.05 vs. Day 0; † p < 0.05 vs. Day 7.
(PDF)

S7 Table Receiver operating characteristics analyses of lung biomarkers and clinical parameters at inclusion to predict weaning from oxygen at Day 28. Definition of abbreviations: AUC = area under the curve; CI = confidence interval; Se = sensitivity; Sp = specificity; KL-6 = Krebs von den Lungen-6; sRAGE = soluble receptor of advanced glycation end-products; CC16 = Club cell protein 16; Ang-2 = Angiopoietin-2; sCD146 = soluble CD146; LDH = lactate dehydrogenase; BMI = body mass index; SOFA = Sequential Organ Failure Assessment. Measurements were performed within the first 48 h of hospital admission in 54 COVID-19 patients. The criterion was determined by the Youden index method. Boldface type indicates statistical significance.
(PDF)

S8 Table Receiver operating characteristics analyses of lung biomarkers and clinical parameters at inclusion to predict weaning from oxygen at Day 60. Definition of abbreviations: AUC = area under the curve; CI = confidence interval; Se = sensitivity; Sp = specificity; KL-6 = Krebs von den Lungen-6; sRAGE = soluble receptor of advanced glycation end-products; CC16 = Club cell protein 16; Ang-2 = Angiopoietin-2; sCD146 = soluble CD146; LDH = lactate dehydrogenase; BMI = body mass index; SOFA = Sequential Organ Failure Assessment. Measurements were performed within the first 48 h of hospital admission in 54 COVID-19 patients. The criterion was determined by the Youden index method. Boldface type indicates statistical significance.
(PDF)
