# Supplementary material for: Circulating biomarkers of bronchoalveolar injury help predict the need for mechanical ventilation in patients with moderate to severe COVID-19 pneumonia: A prospective cohort study
Source: PLoS One. 2026 Jun 29;21(6):e0337792. doi: 10.1371/journal.pone.0337792 (PMC13313340; doi:10.1371/journal.pone.0337792)
Supplement: S1 Appendix — (PDF) [file pone.0337792.s001.pdf]

Sample size formula

The required sample size was determined based on the objective of estimating the proportion of patients requiring mechanical ventilation (MV) with a pre-specified precision. We used the formula for a proportion in a finite population:

$$\mathrm{n} = \frac{N \times X}{X + N - 1}$$

Where

$$X = Z\alpha/2^2 \times p \times (1 - p) \,/\, MOE^2$$

Calculation parameters:

- Expected proportion of patients requiring MV (*p*): 30% (based on early reports).
- Margin of error (MOE): 10%.
- Confidence level (1 − α): 90% ($Z_{\alpha/2}$ = 1.645).
- Estimated source population (*N*): 1000.

Based on these parameters, the minimum required sample size was 54 subjects.

Reference:

Naouri D, Vuagnat A, Beduneau G, Dres M, Pham T, Mercat A, et al. Trends in clinical characteristics and outcomes of all critically ill COVID-19 adult patients hospitalized in France between March 2020 and June 2021: a national database study. Ann Intensive Care. 2023;13: 2. doi:10.1186/s13613-022-01097-3
