# Supplementary material for: Circulating biomarkers of bronchoalveolar injury help predict the need for mechanical ventilation in patients with moderate to severe COVID-19 pneumonia: A prospective cohort study
Source: PLoS One. 2026 Jun 29;21(6):e0337792. doi: 10.1371/journal.pone.0337792 (PMC13313340; doi:10.1371/journal.pone.0337792)
Supplement: S1 Table — Definition of abbreviations: HFOT = high flow oxygen therapy; MV = mechanical ventilation. COVID-19 subgroups were defined according to the maximal level of respiratory support received during hospitalization on the World Health Organization clinical progression scale (WHO-CPS). Data are expressed as number (%). Statistics were performed with Chi-2 test or Fisher’s exact test. (PDF) [file pone.0337792.s004.pdf]

Timing of Day 0 blood sampling	COVID-19			P value
	Oxygen	HFOT	MV	
No. of subjects	18	13	23	
On the day of hospital admission, n (%)	0	0	2 (9)	0.502
On the 1st day following hospital admission, n (%)	10 (56)	10 (77)	12 (52)	0.323
On the 2nd day following hospital admission, n (%)	8 (44)	3 (23)	9 (39)	0.482
