# Supplementary material for: Circulating biomarkers of bronchoalveolar injury help predict the need for mechanical ventilation in patients with moderate to severe COVID-19 pneumonia: A prospective cohort study
Source: PLoS One. 2026 Jun 29;21(6):e0337792. doi: 10.1371/journal.pone.0337792 (PMC13313340; doi:10.1371/journal.pone.0337792)
Supplement: S2 Table — Definition of abbreviations: HFOT = high flow oxygen therapy; MV = mechanical ventilation; sRAGE = soluble receptor of advanced glycation end-products; sCD146 = soluble CD146. Data are presented as median [interquartile range: 25–75%]. Measurements in COVID-19 patients were performed within the first 48 h of hospital admission. Statistical analyses were performed with the Kruskal-Wallis test and post-hoc multiple comparisons with the Conover test. Boldface type indicates statistical significance. * p < 0.05 vs. controls; † p < 0.05 vs. oxygen group; ‡ p < 0.05 vs. HFOT group. (PDF) [file pone.0337792.s005.pdf]

Variables, units	Controls	COVID-19			P value
		Oxygen	HFOT	MV	
No. of subjects	20	18	13	23	
Krebs von den Lungen-6, U/mL	274<br>[200–384]	402<br>[248–613] *	406<br>[287–638] *	614<br>[460–851] *†	**<0.001**
sRAGE, pg/mL	1505<br>[910–1876]	3198<br>[1748–4127] *	2780<br>[2187–4924] *	8026<br>[4000–13251] *†‡	**<0.001**
Club cell protein 16, ng/mL	28.5<br>[19.5–34.5]	15<br>[11–19] *	20<br>[13.8–25] *	27<br>[14.8–43.8] †‡	**0.008**
Angiopoietin-2, pg/mL	2061<br>[1645–2464]	2349<br>[1743–2799]	2648<br>[1989–3686]	2819<br>[2032–4320] *	**0.040**
sCD146, ng/mL	318<br>[238–366]	218<br>[184–245] *	187<br>[160–215] *	213<br>[166–276] *	**<0.001**
