# Supplementary material for: Circulating biomarkers of bronchoalveolar injury help predict the need for mechanical ventilation in patients with moderate to severe COVID-19 pneumonia: A prospective cohort study
Source: PLoS One. 2026 Jun 29;21(6):e0337792. doi: 10.1371/journal.pone.0337792 (PMC13313340; doi:10.1371/journal.pone.0337792)
Supplement: S3 Table — Definition of abbreviations: HFOT = high flow oxygen therapy; MV = mechanical ventilation; HU = Hounsfield unit. COVID-19 subgroups were defined according to the maximal level of respiratory support received during hospitalization on the World Health Organization clinical progression scale (WHO-CPS). Data are presented as median [interquartile range: 25–75%]. Statistical analyses were performed with the Kruskal-Wallis test and post-hoc multiple comparisons with the Conover test for continuous variables. Boldface type indicates statistical significance. * p < 0.05 vs. oxygen group; † p < 0.05 vs. HFOT group. (PDF) [file pone.0337792.s006.pdf]

Variables, units	Oxygen	HFOT	MV	P value
No. of subjects	18	13	23	
**CT-related parameters at inclusion**				
Mean total HU	-683 [-725;-601]	-613 [-671;-524] *	-481 [-614;-368] *†	**<0.001**
Opacity score (0–20)	7 [5–8]	10 [7–15] *	16 [11–18] *†	**<0.001**
Percentage of opacity (0–100)	17 [12–35]	30 [22–48]	61 [32–72] *†	**<0.001**
Percentage of high opacity (0–100)	4 [2–8]	8 [4–15] *	16 [8–24] *†	**0.002**
**Laboratory tests at inclusion**				
Leukocytes, $\times 10^9$/L	6.4 [4.5–8.6]	8.1 [4.7–11.3]	9.7 [7.5–14.6] *	**0.009**
Neutrophils, $\times 10^9$/L	5.2 [2.9–6.9]	7.5 [3.9–9.8]	9 [6.5–12.3] *	**0.001**
Lymphocytes, $\times 10^9$/L	1 [0.8–1.3]	0.8 [0.6–1.1]	0.5 [0.4–0.7] *†	**0.007**
Neutrophil/lymphocyte ratio	4.8 [3.3–7.4]	8.1 [4.8–12.9] *	13.6 [8.6–27.3] *†	**<0.001**
Platelets, $\times 10^9$/L	239 [191–274]	268 [202–302]	215 [188–285]	0.665
D-dimer, µg/mL	0.92 [0.55–1.36]	0.82 [0.59–1.20]	1.25 [0.96–3.41] *†	**0.007**
C-reactive protein, mg/L	94 [38–139]	142 [30–179]	191 [120–229] *†	**<0.001**
Ferritin, µg/L	761 [492–1080]	746 [468–1272]	1255 [791–1831] *†	**0.010**
Lactate dehydrogenase, U/L	319 [248–418]	345 [315–517]	572 [417–663] *†	**<0.001**
Creatinine, µmol/L	61 [52–83]	62 [55–66]	70 [62–96]	0.074
