# Supplementary material for: Circulating biomarkers of bronchoalveolar injury help predict the need for mechanical ventilation in patients with moderate to severe COVID-19 pneumonia: A prospective cohort study
Source: PLoS One. 2026 Jun 29;21(6):e0337792. doi: 10.1371/journal.pone.0337792 (PMC13313340; doi:10.1371/journal.pone.0337792)
Supplement: S4 Table — Definition of abbreviations: BMI = body mass index; SOFA = Sequential Organ Failure Assessment; sRAGE = soluble receptor for advanced glycation end-products; sCD146 = soluble CD146. Data are expressed as number (percentage) or median [interquartile range: 25–75%]. Statistical analyses were performed with the Mann-Whitney U test for continuous variables and the Chi-square test or Fisher’s exact test for categorical variables. Boldface type indicates statistical significance. (PDF) [file pone.0337792.s007.pdf]

Variables, units	**Non-MV**	**MV**	**P value**
No. of subjects	31	23	
Age, yrs	59 [49–72]	66 [57–71]	0.181
Male, n (%)	22 (71)	19 (83)	0.327
BMI, kg/m²	27 [25–30]	31 [29–34]	**0.006**
**Comorbidities**, n (%)			
Diabetes	12 (39)	8 (35)	0.769
Hypertension	10 (32)	13 (57)	0.077
Chronic pulmonary disease	5 (16)	1 (4)	0.177
Chronic heart disease	4 (13)	4 (17)	0.649
Time from first symptom to hospital, days	7 [5–9]	7 [5–8]	0.833
Time from first symptom to inclusion, days	9 [6–11]	9 [7–11]	0.923
SOFA score, at inclusion	2 [2–2]	4 [2–8]	**<0.001**
SpO_2/F_IO_2, at inclusion	294 [183–343]	130 [99–185]	**<0.001**
**Immune therapies in hospital**, n (%)			
Early dexamethasone therapy	27 (87)	23 (100)	0.076
Dexamethasone prior to inclusion	20 (65)	18 (78)	0.279
Rescue methylprednisolone therapy	0	13 (57)	**<0.001**
IL-6 receptor blocker therapy	2 (7)	5 (22)	0.101
IL-6 receptor blocker prior inclusion	1 (3)	1 (4)	0.830
**CT-related parameters at inclusion**			
Mean total HU	-665 [-705;-595]	-545 [-641;-447]	**0.003**
Opacity score (0–20)	8 [5–11]	15 [9–18]	**<0.001**
Percentage of opacity (0–100)	24 [13–36]	61 [32-72]	**<0.001**
Percentage of high opacity (0–100)	6 [3–9]	16 [8–24]	**0.001**
**Laboratory tests at inclusion**			
Leukocytes, $\times 10^9$/L	6.4 [4.7–9.6]	9.7 [7.5–14.6]	**0.006**
Neutrophils, $\times 10^9$/L	5.7 [3.5–8.1]	8.5 [6.5–11.8]	**0.002**
Lymphocytes, $\times 10^9$/L	0.92 [0.68–1.27]	0.54 [0.42–0.75]	**0.002**
Neutrophil/lymphocyte ratio	5.8 [4.1–9.2]	13.6 [8.5–21.7]	**<0.001**
Platelets, $\times 10^9$/L	240 [191–291]	215 [188–285]	0.431
D-dimer, µg/mL	0.90 [0.57–1.21]	1.25 [0.96–3.41]	**0.002**
C-reactive protein, mg/L	104 [37–150]	191 [120–229]	**<0.001**
Ferritin, µg/L	746 [509–1227]	1255 [791–1832]	**0.003**
Lactate dehydrogenase, U/L	342 [267–446]	572 [417–663]	**<0.001**
Creatinine, µmol/L	62 [52–73]	70 [62–96]	**0.022**
**Biomarker levels at inclusion**			
Krebs von den Lungen-6, U/mL	406 [262–634]	614 [460–851]	**0.026**
sRAGE, pg/mL	3033 [1789–4526]	8026 [4000–13251]	**<0.001**
Club cell protein 16, ng/mL	16 [11.3–24]	27 [14.8–43.8]	**0.011**
Angiopoietin-2, pg/mL	2476 [1857–3274]	2819 [2032–4320]	0.122
sCD146, ng/mL	193 [177–239]	213 [166–276]	0.564
