# Supplementary material for: Circulating biomarkers of bronchoalveolar injury help predict the need for mechanical ventilation in patients with moderate to severe COVID-19 pneumonia: A prospective cohort study
Source: PLoS One. 2026 Jun 29;21(6):e0337792. doi: 10.1371/journal.pone.0337792 (PMC13313340; doi:10.1371/journal.pone.0337792)
Supplement: S5 Table — Definition of abbreviations: AUC = area under the curve; CI = confidence interval; Se = sensitivity; Sp = specificity; KL-6 = Krebs von den Lungen-6; sRAGE = soluble receptor of advanced glycation end-products; CC16 = Club cell protein 16; Ang-2 = Angiopoietin-2; sCD146 = soluble CD146; LDH = lactate dehydrogenase; BMI = body mass index; SOFA = Sequential Organ Failure Assessment. Measurements were performed within the first 48 h of hospital admission in 54 COVID-19 patients. The criterion was determined by the Youden index method. Boldface type indicates statistical significance. (PDF) [file pone.0337792.s008.pdf]

Variables, units	Criterion	AUC	95% CI	Se	Sp	P value
KL-6, U/mL	>414	0.679	0.538–0.799	78.3	58.1	**0.016**
sRAGE, pg/mL	>5449	0.786	0.653–0.886	69.6	90.3	**<0.001**
CC16, ng/mL	>26	0.703	0.564–0.820	52.2	87.1	**0.007**
Ang-2, pg/mL	>2687	0.624	0.482–0.752	60.9	64.5	0.116
sCD146, ng/mL	>256.5	0.546	0.405–0.682	34.8	83.9	0.581
CRP, mg/L	>171	0.766	0.631–0.870	69.6	83.9	**<0.001**
Ferritin, µg/L	>718.5	0.743	0.606–0.852	91.3	48.4	**<0.001**
D-dimer, µg/mL	>0,95	0.752	0.616–0.860	78.3	64.5	**<0.001**
LDH, U/L	>496	0.810	0.680–0.904	65.2	83.9	**<0.001**
Creatinine, µmol/L	>60	0.683	0.542–0.803	82.6	48.4	**0.015**
NLR	>9.4	0.836	0.710–0.923	73.9	77.4	**<0.001**
Mean HU total	>-568	0.743	0.603–0.854	59.1	86.7	**<0.001**
Opacity level	>8	0.778	0.645–0.880	82.6	64.5	**<0.001**
Opacity, %	>30.4	0.790	0.657–0.889	78.3	71	**<0.001**
High opacity, %	>7.8	0.763	0.628–0.868	78.3	67.7	**<0.001**
BMI, kg/m²	>29.1	0.719	0.581–0.833	73.9	71	**0.003**
SOFA score	>3	0.818	0.689–0.910	52.2	100	**<0.001**
SpO_2/F_IO_2	≤233	0.861	0.740–0.940	95.7	67.7	**<0.001**
