# Supplementary material for: Circulating biomarkers of bronchoalveolar injury help predict the need for mechanical ventilation in patients with moderate to severe COVID-19 pneumonia: A prospective cohort study
Source: PLoS One. 2026 Jun 29;21(6):e0337792. doi: 10.1371/journal.pone.0337792 (PMC13313340; doi:10.1371/journal.pone.0337792)
Supplement: S6 Table — Definition of abbreviations: MV = mechanical ventilation; KL-6 = Krebs von den Lungen-6; sRAGE = soluble receptor of advanced glycation end-products; CC16 = Club cell protein 16; Ang-2 = Angiopoietin-2; sCD146 = soluble CD146. Data are presented as median [interquartile range: 25–75%]. Measurements were performed in 44 COVID-19 patients at inclusion (Day 0) and at Day 7 and Day 14 following inclusion. COVID-19 patients were stratified by the need for MV during hospitalization into MV (N = 22) and Non-MV (N = 22) groups. Statistical analyses were performed with the Kruskal-Wallis test and post-hoc multiple comparisons with the Conover test. Boldface type indicates statistical significance. * p < 0.05 vs. Day 0; † p < 0.05 vs. Day 7. (PDF) [file pone.0337792.s009.pdf]

| Variables, units | Group | Time | | | P value within group |
|---|---|---|---|---|---|
| | | Day 0 | Day 7 | Day 14 | |
| KL-6, U/mL | MV | 609 [449–817] | 701 [482–985] | 708 [558–839] | 0.578 |
| | Non-MV | 455 [361–634] | 639 [409–881] | 703 [474–1059] | 0.100 |
| | *P value within time* | 0.159 | 0.453 | 0.907 | |
| sRAGE, pg/mL | MV | 7596 [3904–11860] | 1066 [593–1530] * | 713 [523–1649] * | **<0.001** |
| | Non-MV | 2907 [1769–4659] | 1199 [803–1877] * | 1342 [836–1608] * | **<0.001** |
| | *P value within time* | **0.005** | 0.504 | 0.082 | |
| CC16, ng/mL | MV | 26 [14–43] | 31 [20–52] | 42 [30–79] * | **0.020** |
| | Non-MV | 14 [11–20] | 21 [15–24] | 29 [23–32] *† | **<0.001** |
| | *P value within time* | **0.015** | **0.012** | **0.005** | |
| Ang-2, pg/mL | MV | 2791 [1970–3976] | 4580 [3277–6455] | 3704 [2583–7507] | 0.061 |
| | Non-MV | 2580 [2029–3546] | 2828 [1829–3983] | 2881 [2245–3580] | 0.783 |
| | *P value within time* | 0.725 | **0.009** | 0.074 | |
| sCD146, ng/mL | MV | 204 [164–225] | 148 [134–167] * | 169 [153–193] | **0.024** |
| | Non-MV | 212 [181–238] | 218 [196–245] | 239 [198–291] | 0.154 |
| | *P value within time* | 0.270 | **<0.001** | **<0.001** | |
