# Supplementary material for: Circulating biomarkers of bronchoalveolar injury help predict the need for mechanical ventilation in patients with moderate to severe COVID-19 pneumonia: A prospective cohort study
Source: PLoS One. 2026 Jun 29;21(6):e0337792. doi: 10.1371/journal.pone.0337792 (PMC13313340; doi:10.1371/journal.pone.0337792)
Supplement: S7 Table — Definition of abbreviations: AUC = area under the curve; CI = confidence interval; Se = sensitivity; Sp = specificity; KL-6 = Krebs von den Lungen-6; sRAGE = soluble receptor of advanced glycation end-products; CC16 = Club cell protein 16; Ang-2 = Angiopoietin-2; sCD146 = soluble CD146; LDH = lactate dehydrogenase; BMI = body mass index; SOFA = Sequential Organ Failure Assessment. Measurements were performed within the first 48 h of hospital admission in 54 COVID-19 patients. The criterion was determined by the Youden index method. Boldface type indicates statistical significance. (PDF) [file pone.0337792.s010.pdf]

Variables, units	Criterion	AUC	95% CI	Se	Sp	P value
KL-6, U/mL	≤414	0.594	0.452–0.726	52.8	77.8	0.246
sRAGE, pg/mL	≤5449	0.796	0.664–0.893	83.3	72.2	**<0.001**
CC16, ng/mL	≤21	0.688	0.547–0.807	72.2	66.7	**0.024**
Ang-2, pg/mL	≤2687	0.597	0.455–0.728	61.1	61.1	0.242
sCD146, ng/mL	≤192.8	0.549	0.407–0.684	50.0	66.7	0.570
CRP, mg/L	≤171	0.690	0.549–0.809	75.0	66.7	**0.012**
Ferritin, μg/L	≤718.5	0.702	0.562–0.819	44.4	94.4	**0.006**
D-dimer, μg/mL	≤0.95	0.707	0.567–0.823	58.3	77.8	**0.004**
LDH, U/L	≤344.5	0.715	0.575–0.829	47.2	88.9	**0.003**
Creatinine, μmol/L	≤60	0.628	0.486–0.756	44.4	83.3	0.116
NLR	≤9.5	0.791	0.659–0.890	75.00	72.2	**<0.001**
Mean HU total	≤-553	0.687	0.544–0.809	82.9	58.8	**0.025**
Opacity level	≤16	0.731	0.593–0.843	100.0	38.9	**0.003**
Opacity, %	≤63.2	0.750	0.613–0.858	94.4	50.0	**0.001**
High opacity, %	≤16	0.723	0.584–0.836	83.3	61.1	**0.004**
BMI, kg/m²	≤29.4	0.752	0.615–0.859	72.2	77.8	**<0.001**
SOFA score	≤3	0.736	0.598–0.847	91.7	50.0	**<0.001**
SpO_2/F_IO_2	>209	0.833	0.706–0.920	63.9	94.4	**<0.001**
