# Supplementary material for: Circulating biomarkers of bronchoalveolar injury help predict the need for mechanical ventilation in patients with moderate to severe COVID-19 pneumonia: A prospective cohort study
Source: PLoS One. 2026 Jun 29;21(6):e0337792. doi: 10.1371/journal.pone.0337792 (PMC13313340; doi:10.1371/journal.pone.0337792)
Supplement: S8 Table — Definition of abbreviations: AUC = area under the curve; CI = confidence interval; Se = sensitivity; Sp = specificity; KL-6 = Krebs von den Lungen-6; sRAGE = soluble receptor of advanced glycation end-products; CC16 = Club cell protein 16; Ang-2 = Angiopoietin-2; sCD146 = soluble CD146; LDH = lactate dehydrogenase; BMI = body mass index; SOFA = Sequential Organ Failure Assessment. Measurements were performed within the first 48 h of hospital admission in 54 COVID-19 patients. The criterion was determined by the Youden index method. Boldface type indicates statistical significance. (PDF) [file pone.0337792.s011.pdf]

Variables, units	**Criterion**	**AUC**	**95% CI**	**Se**	**Sp**	**p value**
KL-6, U/mL	≤492	0.568	0.426–0.702	50.0	75.0	0.555
sRAGE, pg/mL	≤5449	0.659	0.517–0.782	69.6	62.5	0.155
CC16, ng/mL	≤21	0.738	0.600–0.848	65.2	75.0	**0.008**
Ang-2, pg/mL	≤2687	0.606	0.464–0.736	58.7	75.0	0.272
sCD146, ng/mL	≤228.1	0.674	0.533–0.795	69.6	62.5	0.114
CRP, mg/L	≤179	0.668	0.527–0.791	71.7	62.5	0.090
Ferritin, μg/L	≤746.6	0.644	0.502–0.770	43.5	100.0	0.130
D-dimer, μg/mL	≤0.95	0.571	0.429–0.705	50.0	75.0	0.514
LDH, U/L	≤324	0.626	0.484–0.754	32.6	100.0	0.237
Creatinine, μmol/L	≤62	0.659	0.517–0.782	47.8	87.50	0.168
NLR	≤8.1	0.770	0.636–0.874	58.7	100.0	**<0.001**
Mean HU total	>-613	0.508	0.366–0.649	46.7	71.4	0.949
Opacity level	≤8	0.590	0.447–0.722	47.8	75.0	0.447
Opacity, %	≤30.4	0.590	0.447–0.722	54.3	75.0	0.458
High opacity, %	≤38.1	0.527	0.387–0.665	97.8	25.0	0.831
BMI, kg/m²	≤29.8	0.700	0.560–0.817	67.4	75.0	0.100
SOFA score	≤1	0.575	0.598–0.847	15.2	100.0	0.418
SpO_2/F_IO_2	>153	0.796	0.665–0.894	76.1	87.5	**<0.001**
