# Supplementary material for: Circulating biomarkers of bronchoalveolar injury help predict the need for mechanical ventilation in patients with moderate to severe COVID-19 pneumonia: A prospective cohort study
Source: PLoS One. 2026 Jun 29;21(6):e0337792. doi: 10.1371/journal.pone.0337792 (PMC13313340; doi:10.1371/journal.pone.0337792)
Supplement: Attachment — Submitted filename: Review.docx [file pone.0337792.s012.docx]

| Line No. |  |  |
| --- | --- | --- |
| 143-147 and Study flow figure (S1 figure) | In the study flow figure, please mention the numbers of individuals that were excluded using each of the exclusion criteria.  Please mention the ‘do not resuscitate’ and ‘expected lifespan <72 hrs’ separately in the figure. |  |
| 127-135 | The study design could be improved for the outcome it was meant to study.  The study aimed to find out whether a high level of biomarkers (like sRAGE) were associated with a greater risk of need for MV. As such, the study should have taken two groups: one with high sRAGE levels and one with low sRAGE levels and followed these groups to D60. This would have yielded relative risk, which is more appropriate for the outcome. |  |
| 137-159 | References for the guidelines (used for diagnosis and management of patients, used for deciding use of ECMO) used in the study should be cited and added to the reference list |  |
| 156 | Corticosteroids were used as a rescue for lung fibrosis. Please mention the criteria used to define lung fibrosis in these individuals and cite the reference. |  |
| 201-213 | The outcomes that the study was designed for need to be mentioned clearly. |  |
| 216 | The formula used for sample size calculation should be mentioned either in the text or in the supplementary. |  |
| 283 | How many individuals underwent D0 sampling on days 0, 1, and 2 can be mentioned separately. Also, these numbers can be mentioned for each of the subgroups: Oxygen, HFOT, and MV separately; to better evaluate if delayed sampling was more frequent in any of the subgroups. |  |
| 349 | The confidence interval is very close to 1. A question that arises is if the statistically significant difference is also clinically relevant. |  |
| 418 | How many mortalities were noted by D28? |  |
| 419-422 | Only one set of values are mentioned in the text here, although the text talks about sRAGE and CC16 both. |  |
| 425-426 | Please recheck p values for all parameters mentioned here. |  |
| 428 | Why was this cut-off (sRAGE >5449) chosen? Please mention the basis of this cut-off |  |
| 467-478 | Please stick to discussion of the outcomes that the study was planned with. |  |
| 482-489 | These statements do not add much to the discussion and can be dropped. Instead, please discuss the findings of this study compared to what other studies have found. A lot of research has been published since the study time-period of the authors’ research, and it would be better to talk about that. |  |
| 497-498 | Please recheck this statement and correct it: ‘… HU… decreased gradually, indicating a loss of aeration’ |  |
| 498-501 | What was the OR for LDH to determine the need for MV? Please mention this and discuss the comparison of its performance to sRAGE. |  |
| 502-514 | Repetitive and uninformative. Can be deleted. |  |
| 515-528 | More limitations should be discussed.  The confidence intervals are close to 1.  The clinical relevance of the findings cannot be established based on this study.  The study design is not completely appropriate to find out if a high sRAGE predicts need for MV. Instead, two subgroups of COVID-19 patients should be taken. One with high sRAGE levels on day 0 and one with low. Then find out how many required MV over the course of 28 or 60 days. Based on this, find out the RR. |  |
| 518 | Please re-review published data and discuss it |  |
| 521 | Ideally, these individuals should be excluded from the study, irrespective of whether the authors found an impact on the results or not. The endpoint (MV) was achieved before the measurement (blood sample for biomarkers) was performed. This is a major limitation of the study. |  |
| 534-545 | Please stick to major and relevant findings from the study. There are only two main findings from the research and only these should be mentioned in conclusion. The authors must mention the three big limitations of the study, and that the clinical relevance of these findings (in light of a low OR and CIs close to 1) were beyond the scope of this research study and need to be established. |  |
