# Supplementary material for: Circulating biomarkers of bronchoalveolar injury help predict the need for mechanical ventilation in patients with moderate to severe COVID-19 pneumonia: A prospective cohort study
Source: PLoS One. 2026 Jun 29;21(6):e0337792. doi: 10.1371/journal.pone.0337792 (PMC13313340; doi:10.1371/journal.pone.0337792)
Supplement: Attachment — Submitted filename: Response to Reviewers Plos One_Biomarkers_v1.docx [file pone.0337792.s014.docx]

**Editor Comments:**

**There are several limitations mainly to the methodology pertaining to sample size calculation and exclusion criteria. Primary objective and study design are not matching. Please clarify, what was the primary objective for which this study was designed. What was the hypothesis? Criteria for rescue use of corticosteroids missing. Conclusions should be based on with main results/primary objectives.**

**We thank the editor for giving us the opportunity to resubmit our manuscript to the journal *Plos One*. We have addressed all issues raised by the reviewers, and you will find our point-by-point responses below (blue text). We also revised the manuscript accordingly and provided a new version with tracked changes and a final version with changes identified in red font.**

**Reviewer Comments:**

**Reviewer #1:**

1) **Figure S1: In the study flow figure, please mention the numbers of individuals that were excluded using each of the exclusion criteria.**

**We thank the reviewer for this point. We agree that a clear breakdown of exclusion criteria is essential for the transparency of the study. We would like to point out that these numbers were included in Supplementary Figure S1 (at the beginning of the exclusion lines). However, to improve the clarity and readability of the flow chart as suggested, we have reformatted the figure to ensure that the reasons for exclusion and the corresponding patient counts are more immediately apparent. Please find the revised Figure S1 in the updated Supporting Information.**

2) **Line 143-147: Please mention the ‘do not resuscitate’ and ‘expected lifespan <72 hrs’ separately in the figure.**

**As suggested by the reviewer, we have now disaggregated these two exclusion criteria in the revised Figure S1. The 'do not resuscitate' orders and the 'expected lifespan < 72 hours' are now listed as separate items with their respective patient counts, providing better clarity on the exclusion process.**

3) **Line 127-135: The study design could be improved for the outcome it was meant to study. The study aimed to find out whether a high level of biomarkers (like sRAGE) were associated with a greater risk of need for MV. As such, the study should have taken two groups: one with high sRAGE levels and one with low sRAGE levels and followed these groups to D60. This would have yielded relative risk, which is more appropriate for the outcome.**

**We thank the reviewer for this methodological point. We acknowledge that in our cohort, where 43% of patients required mechanical ventilation (MV), the Odds Ratio (OR) derived from our logistic regression is not a direct substitute for the Relative Risk (RR) and likely overestimates it.**

**However, we opted for this design and analysis for several rigorous reasons.**

**Firstly, partitioning patients into 'high' and 'low' sRAGE groups a priori would have required a validated clinical cutoff, which did not exist at the time of study inception. Creating an arbitrary cutoff would lead to a significant loss of statistical power and could introduce bias. Analyzing sRAGE as a continuous variable allows for a more granular understanding of the dose-response relationship.**

**Secondly, our primary goal was to explore the biological spectrum of COVID-19 across all WHO severity stages. A standard cohort analysis using multivariable logistic regression remains a robust and widely accepted method to identify independent predictors of an outcome like MV, even when the outcome is frequent.**

**Thirdly, this approach allows for a direct comparison with existing literature on COVID-19 and biomarkers, where ORs are frequently reported.**

**We have updated our Study Limitations section to explicitly mention that our results are expressed as ORs and should be interpreted as measures of association rather than direct relative risks (Page 31, Lines 563-567):**

**“*Fourth, our study's observational design led us to report Odds Ratios (OR) rather than Relative Risks (RR). Because the incidence of the primary endpoint (MV) was relatively high in our cohort (43%), the OR may overestimate the RR and should be interpreted as a measure of association rather than a direct risk ratio.*”**

4) **Line 137-159: References for the guidelines (used for diagnosis and management of patients, used for deciding use of ECMO) used in the study should be cited and added to the reference list.**

**We thank the reviewer for this remark. As suggested, we have now cited the specific guidelines used for the diagnosis, clinical management, and ECMO decision-making process in the revised manuscript (Page 8, Lines 144-151). The following references have been added to the reference list:**

1. **World Health Organization. *COVID-19 Clinical Management: Living guidance*, 25 January 2021. Geneva: World Health Organization; 2021. (WHO/2019-nCoV/clinical/2021.1).**
2. **Alhazzani W, et al. *Surviving Sepsis Campaign Guidelines on the Management of Adults With Coronavirus Disease 2019 (COVID-19) in the ICU:* *First Update*. Crit Care Med. 2021;49(3):e219-e234. (PMID: 33555780).**

**These guidelines served as the clinical framework for our patient management and the escalation of care, including the criteria for ECMO.**

5) **Line 156: Corticosteroids were used as a rescue for lung fibrosis. Please mention the criteria used to define lung fibrosis in these individuals and cite the reference.**

**We thank the reviewer for this suggestion. As requested, we have clarified the criteria used to define lung fibrosis in our study. These include a worsening of respiratory system compliance (≤40 ml/cmH_2_O), persistent hypoxemia (PaO_2_/F_I_O_2_≤200), and CT features suggestive of lung fibroproliferation. We have added these details and the corresponding references to the revised manuscript (page 8, lines 151-155):**

**« Patients who developed clinical features of lung fibrosis defined by a worsening of respiratory system compliance (≤40 ml/cmH2O), persistent hypoxemia (PaO2/FIO2≤200), and CT features suggestive of lung fibroproliferation received rescue corticosteroid therapy (methylprednisolone, 2 mg/kg/day for 14 days).”**

- **Meduri et al., JAMA 1998 (PMID: 9669790)**
- **Burnham et al., Chest 2014 (PMID: 24722949)**

6) **Line 201-213: The outcomes that the study was designed for need to be mentioned clearly.**

**We agree with the reviewer that the distinction between primary and secondary outcomes should be more explicit. We have restructured the 'Data collection and Outcomes' section to clearly state our endpoints. As indicated in our introduction, the primary outcome was the requirement for mechanical ventilation (MV) during the hospital stay. Secondary outcomes included weaning from oxygen and oxygen free-days at D28 and D60, and hospital survival. We have updated the manuscript to clarify (Page 11, Lines 212-214):**

**“The primary outcome was the requirement for MV during the hospital stay. Secondary outcomes included weaning from oxygen and oxygen-free days at D28 and D60, and hospital survival.”**

7) **Line 216: The formula used for sample size calculation should be mentioned either in the text or in the supplementary.**

**As suggested by the reviewer, we have detailed the sample size calculation in the supplementary “S1 Appendix”:**

**“The required sample size was determined based on the objective of estimating the proportion of patients requiring mechanical ventilation (MV) with a pre-specified precision. We used the formula for a proportion in a finite population: n = N*X / (X + N - 1)**

**where X = Z_α/2_^2^ *p* (1-p) / MOE^2^**

**Based on an expected proportion of patients requiring mechanical ventilation of 30 %, a margin of error of 10%, a confidence level of 90%, and an estimated source population of 1000 subjects, fifty-four subjects were needed to accurately estimate the true population proportion.”**

8) **Line 283: How many individuals underwent D0 sampling on days 0, 1, and 2 can be mentioned separately. Also, these numbers can be mentioned for each of the subgroups: Oxygen, HFOT, and MV separately; to better evaluate if delayed sampling was more frequent in any of the subgroups.**

**We thank the reviewer for this relevant suggestion. As requested, we have provided a detailed breakdown of the timing for D0 sampling (Day 0, Day 1, and Day 2 post-admission) for each respiratory subgroup (Oxygen, HFOT, and MV).**

**These data have been added to the Supporting Information as “S1 Table”. As shown in this table, the distribution of sampling timing was consistent across all subgroups, suggesting that delayed sampling was not more frequent in any particular category. This ensures that the baseline biomarker levels are comparable between the severity groups.**

9) **Line 349: The confidence interval is very close to 1. A question that arises is if the statistically significant difference is also clinically relevant.**

**We thank the reviewer for this comment. We agree that the reported Odds Ratio (OR) for sRAGE appeared close to 1, which was primarily due to the small increment used for the analysis (100 pg/mL). Given that sRAGE levels in our cohort varied across a wide range (from 292 to 17500 pg/mL), an increment of 100 pg/mL does not fully capture the clinical impact of larger biological variations.**

**To better reflect the clinical relevance, we have updated the analysis using an increment of 1000 pg/mL. The revised independent risk factors for MV are now:**

- **sRAGE (OR per 1000 pg/mL increase 1.316; 95% CI [1.040–1.667]; p=0.022)**
- **SpO_2_/F_I_O_2_ ratio (OR 0.984; 95% CI [0.970–0.998]; p=0.008).**

**With this larger increment, the OR demonstrates a 32% increase in the risk of mechanical ventilation for every 1000 pg/mL increase in sRAGE. This adjustment more clearly illustrates the clinical significance of sRAGE as a predictor in our model. We have updated Table 2 (Page 20) and the corresponding text in the whole manuscript accordingly.**

10) **Line 418: How many mortalities were noted by D28?**

**We thank the reviewer for this question. In our cohort, 3 patients (5.6%) had died by Day 28. To provide this information to the readers without burdening the main text, we have added a row for '28-day mortality' in Table 1 (under the Clinical Outcomes section). We believe this adds valuable context regarding the overall survival of the study population.**

11) **Line 419-422: Only one set of values are mentioned in the text here, although the text talks about sRAGE and CC16 both.**

**We apologize for the lack of clarity in this sentence. Our intention was to highlight that sRAGE was the best predictor for weaning at D28, while CC16 was the best predictor at D60.**

**We have reworded the sentence in the 'Results' section to provide the specific AUC values for each biomarker as requested (Page 24, Lines 413-416):**

**“At inclusion, sRAGE achieved the highest AUC to predict weaning from oxygen at D28 (AUC 0.796 [95% CI: 0.653–0.886]; p<0.001), while CC16 showed the best performance for weaning at D60 (AUC 0.738 [95% CI: 0.600–0.848]; p=0.008) (S7 and S8 Tables).”**

12) **Line 425-426: Please recheck p values for all parameters mentioned here.**

**We thank the reviewer for this suggestion. We have carefully rechecked the coefficients and p-values for the Cox model. Based on the beta coefficient (β = -0,000105) and its standard error (*SE* = 0,00005356), the p-value is confirmed at 0.050.**

**To improve clinical interpretability and maintain consistency with the logistic regression analysis, we have updated the sRAGE increment to 1000 pg/mL. The revised parameters are:**

- **HR: 0.900; 95% CI [0.811–0.999]; p=0.050)**

**While the upper bound of the confidence interval remains close to 1, this independent association is consistent with our other findings. Furthermore, the robust association observed in the categorical analysis (sRAGE ≥ 5449 pg/mL, HR 0.24; 95% CI [0.12–0.48]; p<0.001) reinforces the role of sRAGE as a significant predictor of weaning from oxygen.**

**We have updated the corresponding text in the Results section accordingly (Page 24, Lines 418-419).**

13) **Line 428: Why was this cut-off (sRAGE >5449) chosen? Please mention the basis of this cut-off**

**We apologize for the lack of clarity regarding the selection of this threshold. As described in the 'Statistical Analysis' section of the Methods, the sRAGE cut-off of 5449 pg/mL was determined using the Youden Index method to identify the optimal balance between sensitivity and specificity for predicting oxygen weaning at Day 28.**

**This specific cut-off yielded a sensitivity of 83.3% and a specificity of 72.2% (AUC 0.796, p<0.001), representing the point on the ROC curve that maximizes the biochemical signal for this outcome. We have updated the Results section to explicitly mention the basis and the performance of this cut-off (Page 25, Line 421-424):**

**“Based on the optimal threshold determined by the Youden Index (≥5449 pg/mL; sensitivity 83.3%, specificity 72.2%), COVID-19 patients with sRAGE levels above this cut-off at inclusion had a lower probability of weaning from oxygen at D28…”.**

14) **Line 467-478: Please stick to discussion of the outcomes that the study was planned with.**

**We have revised this paragraph to more clearly highlight our original findings.**

**While the initial low levels of CC16 at admission have been previously described, the sustained and marked increase we observed at Day 14 in patients under mechanical ventilation is a novel observation. We believe this delayed kinetic is important to mention as it likely reflects the persistence of lung epithelial damage in these patients. The text has been updated to emphasize this contribution (Page 27, Line 468-476).**

**“While this paradoxical initial decrease has been reported previously [54,55], our study uniquely shows a progressive and marked increase in CC16 levels up to D14 specifically in patients under MV. This sustained elevation of CC16, occurring while sRAGE levels have already normalized, suggests that CC16 might track different pathological processes, such as persistent bronchoalveolar barrier disruption or secondary injury associated with prolonged mechanical ventilation [54,56]. Our data suggest that these biomarkers provide complementary rather than redundant temporal information regarding the evolution of lung injury in COVID-19.”**

15) **Line 482-489: These statements do not add much to the discussion and can be dropped. Instead, please discuss the findings of this study compared to what other studies have found. A lot of research has been published since the study time-period of the authors’ research, and it would be better to talk about that.**

**We appreciate the reviewer’s suggestion to contextualize our findings with recent literature. However, we would like to clarify that sCD146 remains an emerging and sparsely investigated biomarker in COVID-19. To our knowledge, only one pilot study (Syed et al., 2021) has reported its variations, showing an increase in plasma levels, which directly contrasts with our observation of decreased serum levels.**

**We believe it is crucial to maintain this discussion because our results reveal a novel and unexpected kinetic. Rather than a simple reflection of activation, sCD146 appears to follow a more complex biological regulation. Following your advice, we have significantly revised this paragraph to:**

- **Highlight the scarcity of data on sCD146 in recent COVID-19 research.**
- **Provide a mechanistic framework (downregulation, impaired shedding, or degradation) to explain the observed decrease.**
- **Emphasize the 'mirror kinetics' between sCD146 and Ang-2, showing that both biomarkers consistently track endothelial involvement in MV patients, albeit in opposite directions.**

**We believe these additions provide a much stronger biological foundation for our findings. We have revised the manuscript accordingly (Page 27, Line 477-496):**

**“Our study provides original insights into sCD146, an emerging biomarker of endothelial activation that remains sparsely investigated in COVID-19. While the only available report to date by Syed et al. [38] showed increased plasma sCD146 levels, we observed a significant decrease in serum levels at admission, particularly in patients requiring MV. Despite extensive literature searches, sCD146 remains a rare target in COVID-19 research, but our findings of decreased serum levels are consistent with patterns observed in other acute inflammatory states [57,58].**

**The biological significance of this decrease during the acute phase remains to be fully elucidated. Several non-exclusive mechanisms could be hypothesized: (i) a downregulation of the CD146 receptor expression on the endothelial surface, (ii) a reduction in the proteolytic cleavage (shedding) of its extramembranous part, or (iii) an accelerated intravascular degradation of the soluble form by circulating enzymes.**

**Although our study was not designed to explore these mechanistic pathways, it is noteworthy that sCD146 and Ang-2 exhibited mirrored kinetics over time. In patients requiring MV, both biomarkers showed a gradual and synchronized variation from admission through D14, albeit in opposite directions. This suggests that while their biological regulation differs—likely involving increased release for Ang-2 versus potential downregulation or consumption for sCD146—both markers consistently and concertedly reflect the progression of endothelial involvement in the most severe cases.”**

16) **Line 497-498: Please recheck this statement and correct it: ‘… HU… decreased gradually, indicating a loss of aeration’**

**We are grateful to the reviewer for pointing out this error. We have rechecked the radiological data, and the statement was indeed inverted. In our cohort, the mean HU of the lung parenchyma increased (becoming less negative) with the level of respiratory support, which correctly indicates a loss of lung aeration.**

**The mean HU values were:**

- **Oxygen group: -683 [-725; -601]**
- **HFOT group: -613 [-671; -524]**
- **MV group: -481 [-614; -368]**

**We have corrected the sentence in the Discussion section to accurately reflect these findings (Page 28, Line 498-501):**

**“In this study, AI-based software was used to quantify lung opacities, showing that both the percentage of opacity and the mean HU (Hounsfield Units) increased gradually with the level of respiratory support, indicating a progressive loss of lung aeration.**

17) **Line 498-501: What was the OR for LDH to determine the need for MV? Please mention this and discuss the comparison of its performance to sRAGE.**

**As requested by the reviewer, we have expanded the discussion regarding the comparative performance of LDH and sRAGE. While the raw values were presented in Table 2 (Univariate/Multivariate ORs) and Supplemental Table 4 (AUC/Sensitivity/Specificity), we agree that a more detailed comparison in the Discussion section clarifies their respective clinical roles.**

**Although LDH shows a strong correlation with radiological opacities, the performance of sRAGE is characterized by a higher specificity (90.3% vs. 83.9% for LDH) and a larger effect size (OR 1.32 per 1000 pg/mL increase). Furthermore, as shown in the multivariate model (Table 2), only sRAGE and SpO_2_/F_I_O_2_ remained independent predictors of MV, highlighting that sRAGE captures a specific alveolar signal that LDH—as a marker of global cell lysis—does not.**

**We have added these points to the Discussion (Page 28, Line 497-511):**

**“Determining the strength of the relation between a biomarker and the extent of lung injury yields valuable information to compare biomarkers [56]. In this study, AI-based software was used to quantify lung opacities, showing that both the percentage of opacity and the mean HU (Hounsfield Units) increased gradually with the level of respiratory support, indicating a progressive loss of lung aeration. LDH achieved the highest level of correlation with both the percentage of radiological opacity and the severity of hypoxemia. With a slightly lower strength of correlation than LDH, sRAGE was the lung-specific biomarker that exhibited the highest level of correlation with these two parameters. However, when comparing their performance for predicting the need for MV, sRAGE (AUC 0.786, specificity 90.3%) proved to be a more specific indicator than LDH (AUC 0.810, specificity 83.9%). Indeed, only sRAGE and SpO_2_/F_I_O_2_ remained independently associated with the risk of MV in multivariate analysis (Table 2). This suggests that while LDH reflects global lung tissue damage, sRAGE more specifically tracks the alveolar epithelial insult that drives progressive respiratory failure.”**

18) **Line 502-514: Repetitive and uninformative. Can be deleted.**

**We acknowledge the reviewer's concern regarding redundancy. However, we believe this section is essential as it contextualizes sRAGE within the broader landscape of established COVID-19 risk factors. To address the reviewer’s comment, we have streamlined the paragraph by:**

- **Removing the repetition of specific OR values already mentioned in the previous section.**
- **Focusing on the added value of sRAGE when integrated into a comprehensive model including clinical, radiological, and biological variables—a combination rarely explored in existing literature.**
- **Shortening the concluding remarks to focus on the clinical implications for long-term recovery.**

**We have revised the manuscript accordingly (Page 29, Line 512-524):**

**“While risk factors for MV such as age, comorbidities, and standard laboratory markers are well-documented [57–59], few studies have evaluated the independent weight of lung-specific injury biomarkers in models integrating clinical, radiological, and severity scores. In this comprehensive context, sRAGE and the SpO_2_/F_I_O_2_ ratio remained independent predictors of MV in our cohort. Notably, each 1000 pg/mL increase in sRAGE at admission was associated with a 32% increase in the risk of requiring MV. These results extend the findings of Lim et al. [14] by demonstrating that alveolar epithelial damage is a primary driver of respiratory failure, independent of systemic inflammation. Furthermore, the prognostic value of sRAGE extends beyond the acute phase, as admission levels were associated with a lower probability of oxygen weaning at D60. This suggests that the initial magnitude of the epithelial insult dictates not only the immediate need for life support but also the trajectory of lung recovery.”**

19) **Line 515-528: More limitations should be discussed. The confidence intervals are close to 1. The clinical relevance of the findings cannot be established based on this study. The study design is not completely appropriate to find out if a high sRAGE predicts need for MV. Instead, two subgroups of COVID-19 patients should be taken. One with high sRAGE levels on day 0 and one with low. Then find out how many required MV over the course of 28 or 60 days. Based on this, find out the RR.**

**We thank the reviewer for these insightful comments on the limitations and the statistical approach.**

**Regarding the clinical relevance, we believe that the high specificity of sRAGE (90.3%) for predicting MV, its independence from standard clinical score (SOFA) and radiological findings in multivariate analysis, and its association with long-term recovery (D60 oxygen weaning) provide strong evidence of its potential clinical utility. Furthermore, to address the reviewer's concern regarding confidence intervals (CI) being close to 1, we have revised the expression of sRAGE in our logistic regression models. By using an increment of 1000 pg/mL (instead of the initial 100 pg/mL), which better reflects the clinical range of sRAGE in severe COVID-19, the effect size is now more clearly demonstrated. As shown in Table 2, the OR for sRAGE is 1.32 with a 95% CI of [1.04–1.67] (p=0.022). This adjustment confirms that the association is robust and that the lower bound of the CI is now well above the unit, reinforcing the statistical reliability of sRAGE as an independent predictor.**

**Regarding the study design and the use of RR, we acknowledge the reviewer’s point. While our study was designed as an observational cohort using logistic regression (yielding Odds Ratios), we agree that a 'high vs low' sRAGE approach is valuable for clinicians. Although we did not perform a prospective trial based on a pre-defined sRAGE cut-off, our ROC-curve-derived cut-off and the subsequent multivariate model aim to achieve a similar goal: identifying a threshold of alveolar injury that dictates prognosis.**

**We have expanded the 'Limitations' section to address the narrow confidence intervals and the distinction between OR and RR in our high-incidence cohort (Page 31, Line 559-573):**

**“Third, the study was conducted in a single center with a limited sample size, which resulted in relatively narrow confidence intervals near the unit for some predictors. While statistically significant, these findings should be interpreted with caution and require validation in larger, multicentric cohorts. Fourth, our study's observational design led us to report Odds Ratios (OR) rather than Relative Risks (RR). Because the incidence of the primary endpoint (MV) was relatively high in our cohort (43%), the OR may overestimate the RR and should be interpreted as a measure of association rather than a direct risk ratio. Fifth, although we identified sRAGE as a strong independent predictor, its ultimate clinical relevance—particularly for real-time decision-making—remains to be established through prospective trials comparing outcomes in patients stratified by sRAGE levels at admission. Finally, our findings should be interpreted within the context of the first SARS-CoV-2 variants and the standard use of dexamethasone, and their generalizability to more recent viral strains or different therapeutic regimens remains to be confirmed.”**

20) **Line 518: Please re-review published data and discuss it**

**Following the reviewer's suggestion, we have re-evaluated the literature regarding the impact of corticosteroids on lung injury biomarkers. While their effect on systemic inflammation (e.g., CRP) is well-established, data regarding specific lung injury markers remain very limited.**

**We identified two relevant but contradictory studies. Kim et al. (PMID: 34214123) suggested a more pronounced decline in sRAGE and Ang-2 levels in patients treated with dexamethasone. However, this was a retrospective subgroup analysis of a small cohort (n=32) with a major severity bias (80% severe cases in the treated group vs. 8% in the control group), making it impossible to distinguish the pharmacological effect from the natural clinical course of severe disease.**

**In contrast, the more recent study by Slim et al. (PMID: 38643179) specifically designed to evaluate the effect of immunomodulators on endothelial dysfunction, provides more robust data. With larger and more balanced groups regarding mechanical ventilation (73% vs. 96%), they found no significant effect of corticosteroids on Angiopoietin-2 levels during the first 7 days.**

**Consequently, while data for epithelial markers like sRAGE remain scarce, the best available evidence for endothelial markers suggests that corticosteroids do not significantly bias initial biomarker concentrations.**

**Finally, a comprehensive search of the literature did not identify any studies specifically evaluating the short-term impact of corticosteroids on KL-6, CC16, or sCD146 levels in COVID-19 patients. In the absence of established data suggesting a significant pharmacological interference for these specific markers, we maintain that our findings reflect the underlying severity of lung injury at admission.**

**We have updated our Discussion to reflect this lack of established interference, while maintaining that the balanced distribution of treatments between our study groups (MV vs. non-MV) minimizes the risk of bias (Page 30, Line 539-551):**

**“First, most COVID-19 patients (93%) received corticosteroids during their hospital stay, even prior to inclusion in 69% of cases. While these treatments significantly decrease systemic inflammatory markers [60], their impact on lung injury biomarkers remains poorly documented with contradictory findings. In a pre-specified analysis of a multicentric cohort, Angiopoietin-2 levels were not significantly altered by corticosteroid therapy in COVID-19 [Slim et al., PMID: 38643179]. Although another small subgroup analysis suggested a faster decline in sRAGE and Ang-2 under dexamethasone [Kim et al., PMID: 34214123], this finding was limited by a major imbalance in disease severity between groups. To our knowledge, no specific data are currently available regarding the impact of corticosteroids on other lung injury biomarkers such as CC16, KL-6, or sCD146 in this context. Since the proportion of patients receiving corticosteroids prior to inclusion was similar between our MV and non-MV groups, it is unlikely that these treatments biased our results at admission.”**

21) **Line 521: Ideally, these individuals should be excluded from the study, irrespective of whether the authors found an impact on the results or not. The endpoint (MV) was achieved before the measurement (blood sample for biomarkers) was performed. This is a major limitation of the study.**

**We thank the reviewer for this methodological point. We agree that, strictly speaking, a predictive biomarker should be measured before the event occurs. This is precisely why we performed the sensitivity analysis.**

**However, we would like to highlight a crucial finding: excluding these 7 patients actually improves the predictive performance of sRAGE. The AUC for sRAGE increases from 0.786 (all patients) to 0.888 (excluding the 7 patients). The sensitivity increases from 69.6% to 81.2%, while the specificity remains stable at 90.3%. Most importantly, the optimal cut-off identified by the Youden index remains identical (5449 pg/mL) in both scenarios.**

**These results demonstrate that the initiation of MV prior to sampling does not artificially inflate sRAGE levels; rather, it appears to introduce clinical heterogeneity that slightly weakens the overall statistical performance. By keeping these patients in the main analysis, we chose a more conservative and "real-world" approach, but the robustness of sRAGE as a marker of severe lung injury is even more evident when they are excluded.**

**We have now clarified these points in the Results and Discussion sections to reassure the reader about the absence of an 'incorporation bias' (Page 31, Line 551-559):**

**“Second, MV was initiated prior to the first blood sample in seven of the 23 patients requiring MV. While this could theoretically influence biomarker levels, a sensitivity analysis excluding these seven patients actually yielded a higher AUC for sRAGE (0.888 vs. 0.786) and improved sensitivity (81.2% vs. 69.6%), with an identical optimal cut-off. This suggests that sRAGE levels primarily reflect the severity of the underlying alveolar insult rather than the impact of mechanical ventilation itself. However, we acknowledge that for strict predictive modeling, future studies should ensure that all samples are collected prior to any escalation in respiratory support level.”**

22) **Line 534-545: Please stick to major and relevant findings from the study. There are only two main findings from the research and only these should be mentioned in conclusion. The authors must mention the three big limitations of the study, and that the clinical relevance of these findings (in light of a low OR and CIs close to 1) were beyond the scope of this research study and need to be established.**

**We thank the reviewer for this insightful comment. We have revised the conclusion to focus on the two primary findings while streamlining the discussion of limitations. We particularly highlighted the unique early and transient kinetic profile of sRAGE compared to other biomarkers. Regarding the reviewer's concern about the clinical relevance, we have clarified that the association for sRAGE is robust when using 1000 pg/mL increments (OR 1.32 [1.04–1.67]), although we agree that validation in larger cohorts is essential before clinical implementation. The Discussion section has been modified as follows (Page 33, Line 588-599):**

**”** **In conclusion, this study identifies sRAGE levels at admission and the SpO2/FiO2 ratio as independent predictors of the need for mechanical ventilation in patients hospitalized for COVID-19 pneumonia. A key finding is the early and transient elevation of sRAGE, which distinguishes it from other lung injury biomarkers that showed delayed or less discriminative alterations. While sRAGE demonstrates a robust association with the need for ventilatory support (OR 1.32 per 1000 pg/mL increment), these findings should be interpreted within the context of a single-center study with a limited sample size and the specific era of early SARS-CoV-2 variants and dexamethasone therapy. Furthermore, given the high incidence of the primary endpoint, the use of Odds Ratios rather than Relative Risks may overestimate the association. Ultimately, the clinical relevance of sRAGE for real-time decision-making remains to be established through larger, multicentric prospective trials.”**

**Reviewer #2:**

1) **What was the method of sampling? Consecutive/Random? Need to be specified as only 54 patient have been included in 6 months**

**We thank the reviewer for this important point. Patients were consecutively screened for inclusion. The inclusion of 54 patients over a 6-month period reflects our stringent eligibility criteria, which were strictly applied to ensure cohort homogeneity.**

**As shown in the flowchart (Figure S1), out of 71 screened patients who met the initial inclusion criteria, 17 were subsequently excluded based on the pre-defined exclusion criteria (e.g., chronic respiratory failure, immunosuppressive therapy, or 'do not resuscitate' orders).**

**We have updated the Methods section to explicitly mention the consecutive nature of the screening (Page 7, Line 131-132).**

**“Adult patients with symptomatic SARS-CoV-2 infection were consecutively screened for inclusion in the study if they fulfilled the following criteria:...”**

2) **Why was oxygen requirement not a part of inclusion criterion when all patient included had oxygen requirement in one form or other**

**We thank the reviewer for this observation. During the study period, according to our institutional protocols, only patients with COVID-19 pneumonia requiring oxygen supplementation were admitted to the hospital. Patients with mild disease not requiring oxygen were managed as outpatients and were therefore not screened for this study.**

**Consequently, although 'oxygen requirement' was not explicitly listed as an inclusion criterion in the protocol, it was an intrinsic characteristic of all patients hospitalized for COVID-19 pneumonia in our center. To clarify this for the reader, we have added this detail to the 'Patients selection and management' section (Page 7, Line 136-138):**

**“Of note, according to local institutional protocols, oxygen supplementation was a prerequisite for hospital admission; thus, all included patients required oxygen therapy at the time of inclusion.”**

3) **Why were controls not age matched? Why were COVID patients not requiring oxygen not taken as controls**

**We thank the reviewer for these comments regarding the control group.**

**First, our objective in including a control group was to establish a physiological baseline for the five lung injury biomarkers in individuals with no underlying pulmonary pathology. Using COVID-19 patients not requiring oxygen as controls would not have fulfilled this objective, as even mild SARS-CoV-2 infection can induce a degree of pulmonary inflammation or subclinical epithelial insult that could alter biomarker levels.**

**Second, we included twenty healthy individuals who regularly donated blood to the Etablissement Français du Sang (EFS) to serve as contemporary controls. This source was chosen to ensure that the controls were strictly healthy and free from chronic conditions (such as chronic respiratory failure) that could interfere with biomarker concentrations. Being 'contemporary' ensured that samples were collected and processed under the same conditions as those of the patients. While we acknowledge that the controls were younger than the COVID-19 patients, we focused on ensuring they represented a 'biological gold standard' for lung integrity.**

**We have clarified the rationale for this control group in the Methods section (Page 8, Line 155-160):**

**“Twenty healthy individuals who regularly donated blood to the Etablissement Français du Sang served as contemporary controls to provide physiological baseline values for all biomarkers. These individuals were specifically selected to ensure the absence of chronic respiratory disease or systemic inflammation, representing a 'gold standard' for lung integrity.”**

4) **Why is the S/F ratio Lower in HFOT group as compared to IMV group?**

**We are very grateful to the reviewer for pointing out this inconsistency, which led us to perform a comprehensive audit of all data presented in Table 1.**

**During this quality control process, we identified a transcription error that had caused a shift in some columns. Specifically, the 'SpO_2_/F_I_O_2_' ratio and the 'Length of Hospital Stay' values were affected. The corrected values are as follows:**

- **SpO_2_/F_I_O_2_ ratio: 341 [297–452] for the Oxygen group; 160 [144–233] for the HFOT group; and 130 [99–185] for the MV group. As the reviewer correctly suspected, these corrected values now strictly follow the gradient of clinical severity.**
- **Length of Hospital Stay: The median stay was revised to 10 [8–13] days for the HFOT group and 39 [27–61] days for the MV group (the Oxygen group remains unchanged at 6 [4–8] days).**

**We have thoroughly verified the entire table against our raw database to ensure absolute accuracy. We have updated Table 1 (Page 14) and the corresponding values in the Results section. We sincerely apologize for these transcription oversights and thank the reviewer for helping us improve the precision of our report.**

5) **If immunosuppressive therapies in last 3 months were excluded why was active cancer not excluded?**

**We thank the reviewer for this relevant question. Our exclusion criterion regarding 'immunosuppressive therapies' was specifically designed to minimize interference with the inflammatory response and the circulating levels of the studied biomarkers.**

**In our cohort, only two patients had a history of active cancer (prostate cancer). Importantly, neither was receiving cytotoxic chemotherapy or immunosuppressive drugs. One was under active surveillance and the other was receiving hormonal therapy, neither of which met our predefined criteria for immunosuppression. Thus, their inclusion did not conflict with our study protocol.**

**We believe that excluding all types of cancer, including those not requiring immunosuppressive treatment, would have unnecessarily restricted our sample without providing additional benefit to the study's internal validity.**

**We have clarified this point in the Methods section (Page 7, Line 139-140):**

**“The exclusion criteria were: pregnancy; immunosuppressive therapies within the previous 3 months (including cytotoxic chemotherapy);…”**

6) **Were the patients excluded if they changed from one group to other? Like from oxygen to HFNC?**

**We thank the reviewer for this request for clarification. There was no overlap or transition between groups because patients were classified a posteriori based on the maximum level of respiratory support required during their entire hospital stay.**

**For instance, a patient initially admitted under conventional oxygen therapy who later required HFOT was assigned to the HFOT group. Similarly, any patient requiring invasive mechanical ventilation at any point was assigned to the MV group, regardless of their initial support. This classification ensures that each patient belongs to only one group, representing their peak clinical severity.**

**We have clarified this point in the 'Patients and Study Design' section to avoid any ambiguity (Page 11, Line 206-210):**

**“Respiratory severity was defined by the maximum type of respiratory support required during the hospital stay, according to the World Health Organization clinical progression scale (WHO-CPS) [50]: oxygen by mask or nasal prong [WHO-CPS grade 5]; high flow oxygen therapy (HFOT) [WHO-CPS grade 6]; and MV [WHO-CPS grade 7–9].”**

7) **Discussion should include the present status of these biomarkers in other causes of ARDS**

**We thank the reviewer for this suggestion to broaden the clinical perspective. As requested, we have added a dedicated paragraph to the Discussion section to compare our findings with the current knowledge of these biomarkers in non-COVID-19 Acute Respiratory Distress Syndrome (ARDS).**

**We specifically highlight that sRAGE is a well-established hallmark of alveolar epithelial type I cell injury in classical ARDS. However, recent evidence suggests that sRAGE levels are significantly higher in COVID-19-related ARDS compared to other etiologies (e.g., bacterial pneumonia or extra-pulmonary sepsis), reflecting a particularly intense epithelial insult. We have also enriched the discussion with recent mechanistic data showing that the SARS-CoV-2 nucleocapsid protein acts as a high-affinity ligand for RAGE, which may explain the prominence of this biomarker in our cohort.**

**The Discussion has been modified as follows (Page 29, Line 525-538):**

**“The biomarker profiles observed in our study share similarities with those found in non-COVID-19 ARDS, though some specificities exist. sRAGE is a recognized hallmark of alveolar epithelial type I cell injury. Interestingly, previous studies have shown that sRAGE levels are significantly higher in patients with COVID-19-related ARDS than in those with ARDS from other etiologies (Kapandji et al., 2021; Bhatraju et al., 2021). This may be explained by specific molecular interactions, as the SARS-CoV-2 nucleocapsid (N) protein has recently been identified as a high-affinity ligand for RAGE, potentially magnifying the epithelial injury signal (Xia et al., 2023). Furthermore, circulating levels of the SARS-CoV-2 N-antigen have been positively correlated with sRAGE concentrations, suggesting that systemic viral load is directly linked to the extent of alveolar epithelial insult (Wick et al., 2022). In contrast, markers of endothelial activation (Ang-2) or distal airway injury (CC16) followed patterns in our cohort more similar to those described in classical ARDS, where their elevation is often related to the progression of organ support rather than the initial viral-induced epithelial hit.”**
