# Supplementary material for: Circulating biomarkers of bronchoalveolar injury help predict the need for mechanical ventilation in patients with moderate to severe COVID-19 pneumonia: A prospective cohort study
Source: PLoS One. 2026 Jun 29;21(6):e0337792. doi: 10.1371/journal.pone.0337792 (PMC13313340; doi:10.1371/journal.pone.0337792)
Supplement: Peer review history

# Peer review: Decision Letter 0

**DOI:** 10.1371/journal.pone.0337792.r001

**Authors:** Vijay Hadda

26 Jan 2026

PONE-D-25-60145

Circulating biomarkers of bronchoalveolar injury help predict the need for mechanical ventilation in patients with moderate to severe COVID-19 pneumonia: a prospective cohort study

PLOS One

Dear Dr. Allardet-Servent,

Thank you for submitting your manuscript to PLOS ONE. After careful consideration, we feel that it has merit but does not fully meet PLOS ONE’s publication criteria as it currently stands. Therefore, we invite you to submit a revised version of the manuscript that addresses the points raised during the review process.

Reviewers have submitted their report. There are several limitations mainly to the methodology pertaining to sample size calculation and exclusion criteria. Primary objective and study design are not matching. Please clarify, what was the primary objective for which this study was designed. What was the hypothesis? Criteria for rescue use of corticosteroids missing. Conclusions should be based on with main results/primary objectives.

Please submit your revised manuscript by Mar 12 2026 11:59PM. If you will need more time than this to complete your revisions, please reply to this message or contact the journal office at plosone@plos.org. When you're ready to submit your revision, log on to https://www.editorialmanager.com/pone/ and select the 'Submissions Needing Revision' folder to locate your manuscript file.

Please include the following items when submitting your revised manuscript:

- A letter that responds to each point raised by the academic editor and reviewer(s). You should upload this letter as a separate file labeled 'Response to Reviewers'.
- A marked-up copy of your manuscript that highlights changes made to the original version. You should upload this as a separate file labeled 'Revised Manuscript with Track Changes'.
- An unmarked version of your revised paper without tracked changes. You should upload this as a separate file labeled 'Manuscript'.

If you would like to make changes to your financial disclosure, please include your updated statement in your cover letter. Guidelines for resubmitting your figure files are available below the reviewer comments at the end of this letter.

If applicable, we recommend that you deposit your laboratory protocols in protocols.io to enhance the reproducibility of your results. Protocols.io assigns your protocol its own identifier (DOI) so that it can be cited independently in the future. For instructions see: https://journals.plos.org/plosone/s/submission-guidelines#loc-laboratory-protocols. Additionally, PLOS ONE offers an option for publishing peer-reviewed Lab Protocol articles, which describe protocols hosted on protocols.io. Read more information on sharing protocols at https://plos.org/protocols?utm_medium=editorial-email&utm_source=authorletters&utm_campaign=protocols.

We look forward to receiving your revised manuscript.

Kind regards,

Vijay Hadda, MD

Academic Editor

PLOS One

Journal Requirements:

When submitting your revision, we need you to address these additional requirements.

1.Please ensure that your manuscript meets PLOS ONE's style requirements, including those for file naming. The PLOS ONE style templates can be found at

https://journals.plos.org/plosone/s/file?id=wjVg/PLOSOne_formatting_sample_main_body.pdf and

https://journals.plos.org/plosone/s/file?id=ba62/PLOSOne_formatting_sample_title_authors_affiliations.pdf

2. Please note that your Data Availability Statement is currently missing the repository name. If your manuscript is accepted for publication, you will be asked to provide these details on a very short timeline. We therefore suggest that you provide this information now, though we will not hold up the peer review process if you are unable.

3, If the reviewer comments include a recommendation to cite specific previously published works, please review and evaluate these publications to determine whether they are relevant and should be cited. There is no requirement to cite these works unless the editor has indicated otherwise.

Additional Editor Comments:

There are several limitations mainly to the methodology pertaining to sample size calculation and exclusion criteria. Primary objective and study design are not matching. Please clarify, what was the primary objective for which this study was designed. What was the hypothesis? Criteria for rescue use of corticosteroids missing. Conclusions should be based on with main results/primary objectives.

[Note: HTML markup is below. Please do not edit.]

Reviewers' comments:

Reviewer's Responses to Questions

**Comments to the Author**

1. Is the manuscript technically sound, and do the data support the conclusions?

The manuscript must describe a technically sound piece of scientific research with data that supports the conclusions. Experiments must have been conducted rigorously, with appropriate controls, replication, and sample sizes. The conclusions must be drawn appropriately based on the data presented.

Reviewer #1: Partly

Reviewer #2: Yes

**********

2. Has the statistical analysis been performed appropriately and rigorously?

Reviewer #1: Yes

Reviewer #2: I Don't Know

**********

3. Have the authors made all data underlying the findings in their manuscript fully available?

The PLOS Data policy requires authors to make all data underlying the findings described in their manuscript fully available without restriction, with rare exception (please refer to the Data Availability Statement in the manuscript PDF file). The data should be provided as part of the manuscript or its supporting information, or deposited to a public repository. For example, in addition to summary statistics, the data points behind means, medians and variance measures should be available. If there are restrictions on publicly sharing data—e.g. participant privacy or use of data from a third party—those must be specified.

Reviewer #1: Yes

Reviewer #2: Yes

**********

4. Is the manuscript presented in an intelligible fashion and written in standard English?

PLOS ONE does not copyedit accepted manuscripts, so the language in submitted articles must be clear, correct, and unambiguous. Any typographical or grammatical errors should be corrected at revision, so please note any specific errors here.

Reviewer #1: Yes

Reviewer #2: Yes

**********

5. Review Comments to the Author

Please use the space provided to explain your answers to the questions above. You may also include additional comments for the author, including concerns about dual publication, research ethics, or publication ethics. (Please upload your review as an attachment if it exceeds 20,000 characters)

Reviewer #1: The authors did great work in evaluating a disease at a time when very little was known about it. However, this (study) time period is now five years old. New research has been published since then regarding biomarkers in COVID-19. The review is attached as a separated word file.

Reviewer #2: Following points need to be clarified

What was the method of sampling? Consecutive/Random? Need to be specified as only 54 patient have been included in 6 months

Why was oxygen requirement not a part of inclusion criterion when all patient included had oxygen requirement in one form or other

Why were controls not age matched? Why were COVID patients not requiring oxygen not taken as controls

Why is the S/F ratio Lower in HFOT group as compared to IMV group?

If immunosuppressive therapies in last 3 months were excluded why was active cancer not excluded?

Were the patients excluded if they changed from one group to other? Like from oxygen to HFNC?

Discussion should include the present status of these biomarkers in other causes of ARDS

**********

6. PLOS authors have the option to publish the peer review history of their article (what does this mean?). If published, this will include your full peer review and any attached files.

If you choose “no”, your identity will remain anonymous but your review may still be made public.

**Do you want your identity to be public for this peer review?**  For information about this choice, including consent withdrawal, please see our Privacy Policy.

Reviewer #1: No

Reviewer #2: **Yes:**  Rahul Tyagi

**********

[NOTE: If reviewer comments were submitted as an attachment file, they will be attached to this email and accessible via the submission site. Please log into your account, locate the manuscript record, and check for the action link "View Attachments". If this link does not appear, there are no attachment files.]

To ensure your figures meet our technical requirements, please review our figure guidelines: https://journals.plos.org/plosone/s/figures

You may also use PLOS’s free figure tool, NAAS, to help you prepare publication quality figures: https://journals.plos.org/plosone/s/figures#loc-tools-for-figure-preparation.

NAAS will assess whether your figures meet our technical requirements by comparing each figure against our figure specifications.

Attachment Submitted filename: Review.docx

# Author comment: Author response to Decision Letter 1

**DOI:** 10.1371/journal.pone.0337792.r002

30 Apr 2026

Editor Comments:

There are several limitations mainly to the methodology pertaining to sample size calculation and exclusion criteria. Primary objective and study design are not matching. Please clarify, what was the primary objective for which this study was designed. What was the hypothesis? Criteria for rescue use of corticosteroids missing. Conclusions should be based on with main results/primary objectives.

We thank the editor for giving us the opportunity to resubmit our manuscript to the journal Plos One. We have addressed all issues raised by the reviewers, and you will find our point-by-point responses below (blue text). We also revised the manuscript accordingly and provided a new version with tracked changes and a final version with changes identified in red font.

Reviewer Comments:

Reviewer #1:

1) Figure S1: In the study flow figure, please mention the numbers of individuals that were excluded using each of the exclusion criteria.

We thank the reviewer for this point. We agree that a clear breakdown of exclusion criteria is essential for the transparency of the study. We would like to point out that these numbers were included in Supplementary Figure S1 (at the beginning of the exclusion lines). However, to improve the clarity and readability of the flow chart as suggested, we have reformatted the figure to ensure that the reasons for exclusion and the corresponding patient counts are more immediately apparent. Please find the revised Figure S1 in the updated Supporting Information.

2) Line 143-147: Please mention the ‘do not resuscitate’ and ‘expected lifespan <72 hrs’ separately in the figure.

As suggested by the reviewer, we have now disaggregated these two exclusion criteria in the revised Figure S1. The 'do not resuscitate' orders and the 'expected lifespan < 72 hours' are now listed as separate items with their respective patient counts, providing better clarity on the exclusion process.

3) Line 127-135: The study design could be improved for the outcome it was meant to study. The study aimed to find out whether a high level of biomarkers (like sRAGE) were associated with a greater risk of need for MV. As such, the study should have taken two groups: one with high sRAGE levels and one with low sRAGE levels and followed these groups to D60. This would have yielded relative risk, which is more appropriate for the outcome.

We thank the reviewer for this methodological point. We acknowledge that in our cohort, where 43% of patients required mechanical ventilation (MV), the Odds Ratio (OR) derived from our logistic regression is not a direct substitute for the Relative Risk (RR) and likely overestimates it.

However, we opted for this design and analysis for several rigorous reasons.

Firstly, partitioning patients into 'high' and 'low' sRAGE groups a priori would have required a validated clinical cutoff, which did not exist at the time of study inception. Creating an arbitrary cutoff would lead to a significant loss of statistical power and could introduce bias. Analyzing sRAGE as a continuous variable allows for a more granular understanding of the dose-response relationship.

Secondly, our primary goal was to explore the biological spectrum of COVID-19 across all WHO severity stages. A standard cohort analysis using multivariable logistic regression remains a robust and widely accepted method to identify independent predictors of an outcome like MV, even when the outcome is frequent.

Thirdly, this approach allows for a direct comparison with existing literature on COVID-19 and biomarkers, where ORs are frequently reported.

We have updated our Study Limitations section to explicitly mention that our results are expressed as ORs and should be interpreted as measures of association rather than direct relative risks (Page 31, Lines 563-567):

“Fourth, our study's observational design led us to report Odds Ratios (OR) rather than Relative Risks (RR). Because the incidence of the primary endpoint (MV) was relatively high in our cohort (43%), the OR may overestimate the RR and should be interpreted as a measure of association rather than a direct risk ratio.”

4) Line 137-159: References for the guidelines (used for diagnosis and management of patients, used for deciding use of ECMO) used in the study should be cited and added to the reference list.

We thank the reviewer for this remark. As suggested, we have now cited the specific guidelines used for the diagnosis, clinical management, and ECMO decision-making process in the revised manuscript (Page 8, Lines 144-151). The following references have been added to the reference list:

1. World Health Organization. COVID-19 Clinical Management: Living guidance, 25 January 2021. Geneva: World Health Organization; 2021. (WHO/2019-nCoV/clinical/2021.1).

2. Alhazzani W, et al. Surviving Sepsis Campaign Guidelines on the Management of Adults With Coronavirus Disease 2019 (COVID-19) in the ICU: First Update. Crit Care Med. 2021;49(3):e219-e234. (PMID: 33555780).

These guidelines served as the clinical framework for our patient management and the escalation of care, including the criteria for ECMO.

5) Line 156: Corticosteroids were used as a rescue for lung fibrosis. Please mention the criteria used to define lung fibrosis in these individuals and cite the reference.

We thank the reviewer for this suggestion. As requested, we have clarified the criteria used to define lung fibrosis in our study. These include a worsening of respiratory system compliance (≤40 ml/cmH2O), persistent hypoxemia (PaO2/FIO2≤200), and CT features suggestive of lung fibroproliferation. We have added these details and the corresponding references to the revised manuscript (page 8, lines 151-155):

« Patients who developed clinical features of lung fibrosis defined by a worsening of respiratory system compliance (≤40 ml/cmH2O), persistent hypoxemia (PaO2/FIO2≤200), and CT features suggestive of lung fibroproliferation received rescue corticosteroid therapy (methylprednisolone, 2 mg/kg/day for 14 days).”

- Meduri et al., JAMA 1998 (PMID: 9669790)

- Burnham et al., Chest 2014 (PMID: 24722949)

6) Line 201-213: The outcomes that the study was designed for need to be mentioned clearly.

We agree with the reviewer that the distinction between primary and secondary outcomes should be more explicit. We have restructured the 'Data collection and Outcomes' section to clearly state our endpoints. As indicated in our introduction, the primary outcome was the requirement for mechanical ventilation (MV) during the hospital stay. Secondary outcomes included weaning from oxygen and oxygen free-days at D28 and D60, and hospital survival. We have updated the manuscript to clarify (Page 11, Lines 212-214):

“The primary outcome was the requirement for MV during the hospital stay. Secondary outcomes included weaning from oxygen and oxygen-free days at D28 and D60, and hospital survival.”

7) Line 216: The formula used for sample size calculation should be mentioned either in the text or in the supplementary.

As suggested by the reviewer, we have detailed the sample size calculation in the supplementary “S1 Appendix”:

“The required sample size was determined based on the objective of estimating the proportion of patients requiring mechanical ventilation (MV) with a pre-specified precision. We used the formula for a proportion in a finite population: n = N*X / (X + N - 1)

where X = Zα/22 *p* (1-p) / MOE2

Based on an expected proportion of patients requiring mechanical ventilation of 30 %, a margin of error of 10%, a confidence level of 90%, and an estimated source population of 1000 subjects, fifty-four subjects were needed to accurately estimate the true population proportion.”

8) Line 283: How many individuals underwent D0 sampling on days 0, 1, and 2 can be mentioned separately. Also, these numbers can be mentioned for each of the subgroups: Oxygen, HFOT, and MV separately; to better evaluate if delayed sampling was more frequent in any of the subgroups.

We thank the reviewer for this relevant suggestion. As requested, we have provided a detailed breakdown of the timing for D0 sampling (Day 0, Day 1, and Day 2 post-admission) for each respiratory subgroup (Oxygen, HFOT, and MV).

These data have been added to the Supporting Information as “S1 Table”. As shown in this table, the distribution of sampling timing was consistent across all subgroups, suggesting that delayed sampling was not more frequent in any particular category. This ensures that the baseline biomarker levels are comparable between the severity groups.

9) Line 349: The confidence interval is very close to 1. A question that arises is if the statistically significant difference is also clinically relevant.

We thank the reviewer for this comment. We agree that the reported Odds Ratio (OR) for sRAGE appeared close to 1, which was primarily due to the small increment used for the analysis (100 pg/mL). Given that sRAGE levels in our cohort varied across a wide range (from 292 to 17500 pg/mL), an increment of 100 pg/mL does not fully capture the clinical impact of larger biological variations.

To better reflect the clinical relevance, we have updated the analysis using an increment of 1000 pg/mL. The revised independent risk factors for MV are now:

• sRAGE (OR per 1000 pg/mL increase 1.316; 95% CI [1.040–1.667]; p=0.022)

• SpO2/FIO2 ratio (OR 0.984; 95% CI [0.970–0.998]; p=0.008).

With this larger increment, the OR demonstrates a 32% increase in the risk of mechanical ventilation for every 1000 pg/mL increase in sRAGE. This adjustment more clearly illustrates the clinical significance of sRAGE as a predictor in our model. We have updated Table 2 (Page 20) and the corresponding text in the whole manuscript accordingly.

10) Line 418: How many mortalities were noted by D28?

We thank the reviewer for this question. In our cohort, 3 patients (5.6%) had died by Day 28. To provide this information to the readers without burdening the main text, we have added a row for '28-day mortality' in Table 1 (under the Clinical Outcomes section). We believe this adds valuable context regarding the overall survival of the study population.

11) Line 419-422: Only one set of values are mentioned in the text here, although the text talks about sRAGE and CC16 both.

We apologize for the lack of clarity in this sentence. Our intention was to highlight that sRAGE was the best predictor for weaning at D28, while CC16 was the best predictor at D60.

We have reworded the sentence in the 'Results' section to provide the specific AUC values for each biomarker as requested (Page 24, Lines 413-416):

“At inclusion, sRAGE achieved the highest AUC to predict weaning from oxygen at D28 (AUC 0.796 [95% CI: 0.653–0.886]; p<0.001), while CC16 showed the best performance for weaning at D60 (AUC 0.738 [95% CI: 0.600–0.848]; p=0.008) (S7 and S8 Tables).”

12) Line 425-426: Please recheck p values for all parameters mentioned here.

We thank the reviewer for this suggestion. We have carefully rechecked the coefficients and p-values for the Cox model. Based on the beta coefficient (β = -0,000105) and its standard error (SE = 0,00005356), the p-value is confirmed at 0.050.

To improve clinical interpretability and maintain consistency with the logistic regression analysis, we have updated the sRAGE increment to 1000 pg/mL. The revised parameters are:

• HR: 0.900; 95% CI [0.811–0.999]; p=0.050)

While the upper bound of the confidence interval remains close to 1, this independent association is consistent with our other findings. Furthermore, the robust association observed in the categorical analysis (sRAGE ≥ 5449 pg/mL, HR 0.24; 95% CI [0.12–0.48]; p<0.001) reinforces the role of sRAGE as a significant predictor of weaning from oxygen.

We have updated the corresponding text in the Results section accordingly (Page 24, Lines 418-419).

13) Line 428: Why was this cut-off (sRAGE >5449) chosen? Please mention the basis of this cut-off

We apologize for the lack of clarity regarding the selection of this threshold. As described in the 'Statistical Analysis' section of the Methods, the sRAGE cut-off of 5449 pg/mL was determined using the Youden Index method to identify the optimal balance between sensitivity and specificity for predicting oxygen weaning at Day 28.

This specific cut-off yielded a sensitivity of 83.3% and a specificity of 72.2% (AUC 0.796, p<0.001), representing the point on the ROC curve that maximizes the biochemical signal for this outcome. We have updated the Results section to explicitly mention the basis and the performance of this cut-off (Page 25, Line 421-424):

“Based on the optimal threshold determined by the Youden Index (≥5449 pg/mL; sensitivity 83.3%, specificity 72.2%), COVID-19 patients with sRAGE levels above this cut-off at inclusion had a lower probability of weaning from oxygen at D28…”.

14) Line 467-478: Please stick to discussion of the outcomes that the study was planned with.

We have revised this paragraph to more clearly highlight our original findings.

While the initial low levels of CC16 at admission have been previously described, the sustained and marked increase we observed at Day 14 in patients under mechanical ventilation is a novel observation. We believe this delayed kinetic is important to mention as it likely reflects the persistence of lung epithelial damage in these patients. The text has been updated to emphasize this contribution (Page 27, Line 468-476).

“While this paradoxical initial decrease has been reported previously [54,55], our study uniquely shows a progressive and marked increase in CC16 levels up to D14 specifically in patients under MV. This sustained elevation of CC16, occurring while sRAGE levels have already normalized, suggests that CC16 might track different pathological processes, such as persistent bronchoalveolar barrier disruption or secondary injury associated with prolonged mechanical ventilation [54,56]. Our data suggest that these biomarkers provide complementary rather than redundant temporal information regarding the evolution of lung injury in COVID-19.”

15) Line 482-489: These statements do not add much to the discussion and can be dropped. Instead, please discuss the findings of this study compared to what other studies have found. A lot of research has been published since the study time-period of the authors’ research, and it would be better to talk about that.

We appreciate the reviewer’s suggestion to contextualize our findings with recent literature. However, we would like to clarify that sCD146 remains an emerging and sparsely investigated biomarker in COVID-19. To our knowledge, only one pilot study (Syed et al., 2021) has reported its variations, showing an increase in plasma levels, which directly contrasts with our observation of decreased serum levels.

We believe it is crucial to maintain this discussion because our results reveal a novel and unexpected kinetic. Rather than a simple reflection of activation, sCD146 appears to follow a more complex biological regulation. Following your advice, we have significantly revised this paragraph to:

• Highlight the scarcity of data on sCD146 in recent COVID-19 research.

• Provide a mechanistic framework (downregulation, impaired shedding, or degradation) to explain the observed decrease.

• Emphasize the 'mirror kinetics' between sCD146 and Ang-2, showing that both biomarkers consistently track endothelial involvement in MV patients, albeit in opposite directions.

We believe these additions provide a much stronger biological foundation for our findings. We have revised the manuscript accordingly (Page 27, Line 477-496):

“Our study provides original insights into sCD146, an emerging biomarker of endothelial activation that remains sparsely investigated in COVID-19. While the only available report to date by Syed et al. [38] showed increased plasma sCD146 levels, we observed a significant decrease in serum levels at admission, particularly in patients requiring MV. Despite extensive literature searches, sCD146 remains a rare target in COVID-19 research, but our findings of decreased serum levels are consistent with patterns observed in other acute inflammatory states [57,58].

The biological significance

Attachment Submitted filename: Response to Reviewers Plos One_Biomarkers_v1.docx

# Peer review: Decision Letter 1

**DOI:** 10.1371/journal.pone.0337792.r003

**Authors:** Vijay Hadda, Vijay Hadda

10 Jun 2026

Circulating biomarkers of bronchoalveolar injury help predict the need for mechanical ventilation in patients with moderate to severe COVID-19 pneumonia: a prospective cohort study

PONE-D-25-60145R1

Dear Dr. Allardet-Servent,

We’re pleased to inform you that your manuscript has been judged scientifically suitable for publication and will be formally accepted for publication once it meets all outstanding technical requirements.

Within one week, you’ll receive an e-mail detailing the required amendments. When these have been addressed, you’ll receive a formal acceptance letter and your manuscript will be scheduled for publication.

An invoice will be generated when your article is formally accepted. Please note, if your institution has a publishing partnership with PLOS and your article meets the relevant criteria, all or part of your publication costs will be covered. Please make sure your user information is up-to-date by logging into Editorial Manager at Editorial Manager® and clicking the ‘Update My Information' link at the top of the page. For questions related to billing, please contact billing support.

If your institution or institutions have a press office, please notify them about your upcoming paper to help maximize its impact. If they’ll be preparing press materials, please inform our press team as soon as possible -- no later than 48 hours after receiving the formal acceptance. Your manuscript will remain under strict press embargo until 2 pm Eastern Time on the date of publication. For more information, please contact onepress@plos.org.

Kind regards,

Vijay Hadda, MD

Academic Editor

PLOS One

Additional Editor Comments (optional):

Reviewers' comments:

Reviewer's Responses to Questions

**Comments to the Author**

1. If the authors have adequately addressed your comments raised in a previous round of review and you feel that this manuscript is now acceptable for publication, you may indicate that here to bypass the “Comments to the Author” section, enter your conflict of interest statement in the “Confidential to Editor” section, and submit your "Accept" recommendation.

Reviewer #1: All comments have been addressed

Reviewer #2: All comments have been addressed

**********

2. Is the manuscript technically sound, and do the data support the conclusions?

The manuscript must describe a technically sound piece of scientific research with data that supports the conclusions. Experiments must have been conducted rigorously, with appropriate controls, replication, and sample sizes. The conclusions must be drawn appropriately based on the data presented.

Reviewer #1: Yes

Reviewer #2: Yes

**********

3. Has the statistical analysis been performed appropriately and rigorously?

Reviewer #1: Yes

Reviewer #2: I Don't Know

**********

4. Have the authors made all data underlying the findings in their manuscript fully available?

The PLOS Data policy requires authors to make all data underlying the findings described in their manuscript fully available without restriction, with rare exception (please refer to the Data Availability Statement in the manuscript PDF file). The data should be provided as part of the manuscript or its supporting information, or deposited to a public repository. For example, in addition to summary statistics, the data points behind means, medians and variance measures should be available. If there are restrictions on publicly sharing data—e.g. participant privacy or use of data from a third party—those must be specified.

Reviewer #1: (No Response)

Reviewer #2: Yes

**********

5. Is the manuscript presented in an intelligible fashion and written in standard English?

PLOS ONE does not copyedit accepted manuscripts, so the language in submitted articles must be clear, correct, and unambiguous. Any typographical or grammatical errors should be corrected at revision, so please note any specific errors here.

Reviewer #1: Yes

Reviewer #2: Yes

**********

6. Review Comments to the Author

Please use the space provided to explain your answers to the questions above. You may also include additional comments for the author, including concerns about dual publication, research ethics, or publication ethics. (Please upload your review as an attachment if it exceeds 20,000 characters)

Reviewer #1: The reviewer would like to thank the authors for intensively considering the suggestions and revising the manuscript. There are a few areas of concern, however, and the authors have acceptably defended their choice to retain them as unchanged.

Reviewer #2: The recommended changes have been made and the article has been modified accordingly

Recommended for publication

**********

7. PLOS authors have the option to publish the peer review history of their article (what does this mean?). If published, this will include your full peer review and any attached files.

If you choose “no”, your identity will remain anonymous but your review may still be made public.

**Do you want your identity to be public for this peer review?**  For information about this choice, including consent withdrawal, please see our Privacy Policy.

Reviewer #1: No

Reviewer #2: **Yes:**  Rahul Tyagi

**********

# Editor report: Acceptance letter

**DOI:** 10.1371/journal.pone.0337792.r004

**Authors:** Vijay Hadda, Vijay Hadda

PONE-D-25-60145R1

PLOS One

Dear Dr. Allardet-Servent,

I'm pleased to inform you that your manuscript has been deemed suitable for publication in PLOS One. Congratulations! Your manuscript is now being handed over to our production team.

At this stage, our production department will prepare your paper for publication. This includes ensuring the following:

* All references, tables, and figures are properly cited

* All relevant supporting information is included in the manuscript submission,

* There are no issues that prevent the paper from being properly typeset

You will receive further instructions from the production team, including instructions on how to review your proof when it is ready. Please keep in mind that we are working through a large volume of accepted articles, so please give us a few days to review your paper and let you know the next and final steps.

Lastly, if your institution or institutions have a press office, please let them know about your upcoming paper now to help maximize its impact. If they'll be preparing press materials, please inform our press team within the next 48 hours. Your manuscript will remain under strict press embargo until 2 pm Eastern Time on the date of publication. For more information, please contact onepress@plos.org.

You will receive an invoice from PLOS for your publication fee after your manuscript has reached the completed accept phase. If you receive an email requesting payment before acceptance or for any other service, this may be a phishing scheme. Learn how to identify phishing emails and protect your accounts at https://explore.plos.org/phishing.

If we can help with anything else, please email us at customercare@plos.org.

Thank you for submitting your work to PLOS ONE and supporting open access.

Kind regards,

PLOS ONE Editorial Office Staff

on behalf of

Dr. Vijay Hadda

Academic Editor

PLOS One
